# Brain cells derived from Alzheimer’s disease patients have multiple specific innate abnormalities in energy metabolism

**DOI:** 10.1038/s41380-021-01068-3

**Published:** 2021-04-16

**Authors:** Woo-In Ryu, Mariana K. Bormann, Minqi Shen, Dohoon Kim, Brent Forester, Yeongwon Park, Jisun So, Hyemyung Seo, Kai-C. Sonntag, Bruce M. Cohen

**Affiliations:** 1 Department of Psychiatry, Harvard Medical School, Belmont, MA USA; 2 Basic Neuroscience Division, Harvard Medical School, Belmont, MA USA; 3 Program for Neuropsychiatric Research, McLean Hospital, Harvard Medical School, Belmont, MA USA; 4 Department of Immunology, Tufts University School of Medicine, Boston, MA USA; 5 Mood Disorders Division and Geriatric Psychiatry Research Program, McLean Hospital, Harvard Medical School, Belmont, MA USA; 6 Department of Molecular and Life Sciences, Center for Bionano Intelligence Education and Research, Hanyang University, Ansan, South Korea; 7 Jean Mayer USDA Human Nutrition Research Center on Aging, Tufts University, Boston, MA USA

**Keywords:** Cell biology, Neuroscience, Stem cells, Biochemistry, Diseases

## Abstract

Altered energy metabolism has been implicated both in aging and the pathogenesis of late-onset Alzheimer’s disease (LOAD). However, it is unclear which anomalies are acquired phenotypes and which are inherent and predispose to disease. We report that neural progenitor cells and astrocytes differentiated from LOAD patient-derived induced pluripotent stem cells exhibit multiple inter-related bioenergetic alterations including: changes in energy production by mitochondrial respiration versus glycolysis, as a consequence of alterations in bioenergetic substrate processing and transfer of reducing agents, reduced levels of NAD/NADH, diminished glucose uptake and response rates to insulin (INS)/IGF-1 signaling, decreased INS receptor and glucose transporter 1 densities, and changes in the metabolic transcriptome. Our data confirm that LOAD is a “multi-hit” disorder and provide evidence for innate inefficient cellular energy management in LOAD that likely predisposes to neurodegenerative disease with age. These processes may guide the development and testing of diagnostic procedures or therapeutic agents.

## Introduction

Late-onset Alzheimer’s disease (LOAD), the most common form of dementia, is a neurodegenerative disorder typified by onset after age 65. It is characterized by slowly progressive deterioration and death of neurons whose cause is only partly known [1]. While many factors underlie the risk and development of LOAD, the “amyloid cascade hypothesis” proposes that an accumulation of beta amyloid along with twisted strands of hyperphosphorylated *tau* (tangles) are the primary toxic agents in AD [2]. This hypothesis is best supported for familial or early-onset forms of AD (EOAD), less so for LOAD [3, 4]. Increasing evidence suggests that in LOAD the accumulation of these toxic molecules may not be the sole or initial cause of disease and may instead largely be consequences of other causative factors [3–8]. Notably, a major determinant of LOAD is aging, and many pathological features of LOAD are shared with the normal aging process [9, 10]. In addition, unlike EOAD, which is largely determined by single gene variants, LOAD has multiple genetic and environmental risk determinants. It is a “multi-hit” disorder, the product of many combined interacting risk factors.

A key factor in cellular function and altered in aging is bioenergetics, i.e., the metabolism of various fuel molecules to produce and utilize energy, through glycolysis, mitochondrial respiration, and the pentose phosphate pathway (PPP), which together produce ATP and other essential metabolites. Glycolysis produces pyruvate and lactate from glucose and reduces nicotinamide adenine dinucleotide (NAD), an oxidizing agent involved in redox reactions and mitochondrial electron transfer. Mitochondria primarily metabolize carbohydrates (pyruvate or lactate), ketones, fatty acids, and glutamine, via the Krebs/tricarboxylic/citric-acid cycle (TCA/CAC) and oxidative phosphorylation (OxPhos). The PPP leads to the reduction of NAPD^+^ to NAPDH, used for fatty acid biosynthesis and regeneration of reduced glutathione.

Eukaryotic cells use about 10% glycolysis and 90% OxPhos for energy production, with proportions depending on cell type and metabolic state [11]. The brain consumes about 20% of total body oxygen and utilizes 25% of total body glucose, extracting approximately 50% of oxygen and 10% of glucose from the arterial blood. Aerobic glycolysis is the major mechanism of energy production in the brain with astrocytes consuming about 85% of the glucose utilized brain-wide to generate pyruvate and release lactate [12, 13]. These high energy needs make the brain especially susceptible to energy production and flow disruptions, which could be important considerations in brain aging and neurodegeneration. Mitochondrial functions decrease with age, and many brain disorders, including AD, show evidence of abnormal energy metabolism [9, 10, 14–18].

However, it is unknown whether metabolic pathologies associated with LOAD are inherent or acquired during aging. We recently reported that fibroblasts derived from LOAD patients had impairment in multiple interacting components of bioenergetic metabolism that appeared to be inherent, not acquired, as they were disease related, not age specific [19]. We now report the bioenergetic and metabolic properties of proliferating brain cells, including neural progenitor cells (NPCs) and astrocytes differentiated from LOAD patients’ or AD-unaffected individuals’ induced pluripotent stem cells (iPSCs). NPCs are the earliest progenitors from which all brain cell types develop, and astrocytes play multiple metabolic roles in the brain [12, 13, 20–24]. We show that both LOAD NPCs and astrocytes exhibit multiple altered bioenergetic and related metabolic features suggesting that LOAD is associated with inherent abnormal and insufficient cellular energy management. As seen both in precursor and mature cells, these would be present early and throughout life, consistent with the long course over which LOAD develops [9]. These abnormalities could underlie a predisposition to altered aging as a risk factor in disease development.

## Results

### Generation of iPSCs and differentiation of NPCs and astrocytes

**Fig. 1:**
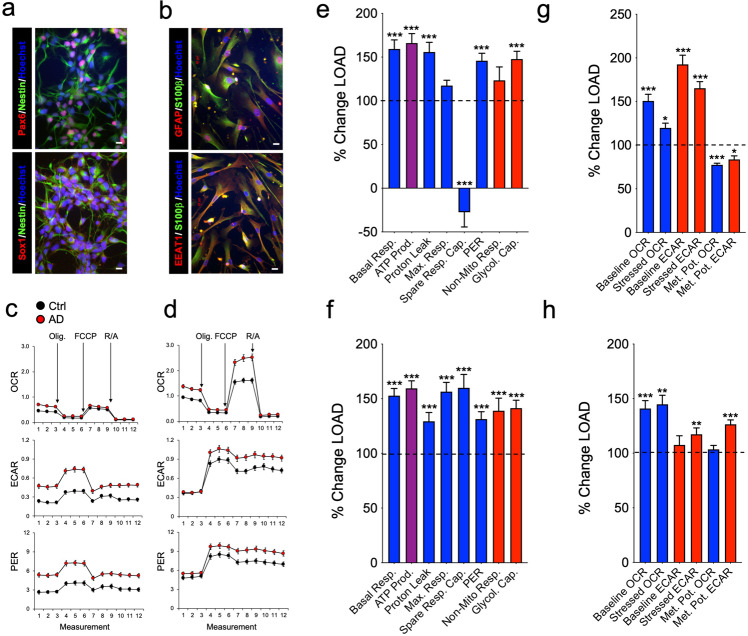
LOAD and Control NPCs and astrocytes exhibit differences in their bioenergetic profiles. **a**, **b** Representative immunocytochemistry of NPCs (C4) expressing PAX6, SOX1, and NESTIN (**a**), and astrocytes (C2) expressing GFAP, S100β, and EEAT1 (**b**). Hoechst was used to stain nuclei. Scale bars, 20 µm. A complete documentation of NPCs and astrocytes is provided in Supplementary Fig. 2c, d. **c**, **d** Profiles of Seahorse XFp Mito Stress Test data for oxidative consumption rates (OCR, pmol/min), extracellular acidification rate (ECAR, mpH/min), and proton efflux rate (PER, pmol H+/min) in LOAD (*n* = 9) and Control NPCs (*n* = 5) (**c**) and LOAD (*n* = 9) and Control astrocytes (*n* = 5) (**d**). LOAD samples are in red and Control samples in black. Arrows indicate injections of specific standard stressors of mitochondrial respiration, including oligomycin (Olig.), carbonyl cyanite-4 (trifluoromethoxy) phenylhydrazone (FCCP), and Rotenone/Antimycin A (R/A). **e**–**g** Calculated values of OCR (blue) and ECAR (red) parameters for NPCs (**e**) and astrocytes (**f**), and results from Cell Energy Phenotype Test Report Generator for NPCs (**g**) and astrocytes (**h**) plotted as percent change LOAD over Control. Data are mean ± SEM from two repeat experiments. *p < 0.1; **p < 0.05; ***p < 0.01 using one-way ANOVA.

iPSCs were induced from LOAD patients’-derived dermal fibroblasts (*n* = 6) or peripheral blood mononucleocytes (PBMCs) (*n* = 3) and from non-demented control subjects’ fibroblasts (*n* = 5) using Sendai virus or RNA reprogramming (Supplementary Table 1). Except one Asian (C1), all donors were Caucasians, and from both sexes with ages 21–91. Two iPSC lines (C1 and C2) were previously published [25, 26]. The remaining lines were characterized using the pluripotent markers OCT4, SOX2, TRA1-60, and TRA1-181 (AD7, AD8, and AD9) and TaqMan hPSC scorecard assays (C3, C4, C5, AD7, AD8, and AD9) (Supplementary Fig. 1a, b). The iPSC lines had multi-lineage differentiation functions in vitro including neural, mesenchymal, and epithelial cells, and formed teratomas in vivo (Supplementary Fig. 1c). During expansion, all iPSC lines had normal karyotypes. Starting at passages 18–20, iPSCs were differentiated to NPCs and astrocytes and characterized by immunocytochemistry (ICC). NPCs expressed the immature markers SOX1, PAX6, and NESTIN. Astrocytes were positive for typical astrocytic markers, including GFAP, S100β, and EEAT1 (GLAST-1), similar to secondary human cortical astrocytes (Fig. 1a, b and Supplementary Fig. 2a–f). Analysis of experimental results from fibroblast- or PBMC-derived iPSC-differentiated NPCs and astrocytes separately showed similar results on outcomes measured demonstrating no influence of the iPSC parental cell types in the descendant cell lines.

### LOAD and Control NPCs and astrocytes exhibit differences in their bioenergetic profiles

Disturbed bioenergetics, including increased mitochondrial oxidative stress and dysfunctional glucose metabolism, have been implicated in the pathogenesis of LOAD [9, 18, 27]. To determine the bioenergetic profiles of NPCs and astrocytes, we analyzed the cell lines in Seahorse XFp Mito Stress Tests, as previously described [19]. Compared to control cells, both LOAD NPCs and astrocytes exhibited increased oxidative consumption rates (OCR), a measure of mitochondrial respiration, and extracellular acidification (ECAR) and proton eflux rates (PER) that are indirect measures of glycolysis (Fig. 1c, d). Further analysis showed that LOAD NPCs and astrocytes had increases of basal and maximal respiration, ATP production, proton leak, non-mitochondrial respiration, and PER, while the spare respiratory capacity (i.e., maximal minus basal respiration as a measure of mitochondrial reserve) was decreased in NPCs and increased in astrocytes (Fig. 1e, f and Supplementary Fig. 3a, b). In addition, glycolysis was increased in LOAD NPCs and astrocytes, while the OCR/ECAR indexes for basal respiration and ATP production, as an indicator of mitochondrial energy output relative to glycolysis, were reduced in LOAD NPCs and increased or unchanged in LOAD astrocytes (Fig. 1e, f and Supplementary Fig. 3b, c).

Additional Seahorse “Cell Energy Phenotype Tests” that evaluated the metabolic potentials for OCR and ECAR by calculating changes after stress conditions relative to baseline [19] showed that in LOAD NPCs both OCR and ECAR baseline and stress levels were increased but not the respective metabolic potentials (percent stress over baseline) (Fig. 1g). In LOAD astrocytes there was an increase in the OCR baseline and stress parameters but not in the metabolic potential, while the ECAR baseline was unchanged and the ECAR metabolic potential increased (Fig. 1h). Furthermore, the Seahorse results showed differences between NPCs and astrocytes with Control and LOAD astrocytes demonstrating overall higher levels of OCR or ECAR parameters than NPCs (Supplementary Fig. 3a–e). Finally, the iPSC-derived Control astrocytes had Seahorse profiles similar to secondary human astrocytes (Supplementary Fig. 4).

In summary, both LOAD NPCs and astrocytes exhibit increased mitochondrial respiration, energy (ATP) output, and elevated glycolytic activity. Importantly, the spare respiratory capacity in LOAD NPCs was reduced indicative of dysfunctional mitochondria. In response to mitochondrial stress, LOAD NPCs showed no respiratory or glycolytic plasticity (metabolic potential), while astrocytes could upregulate glycolysis. Overall, astrocytes had higher bioenergetic rates and higher respiratory capacity than NPCs.

### NAD^+^ synthesis and recycling is reduced in LOAD NPCs and astrocytes

**Fig. 2:**
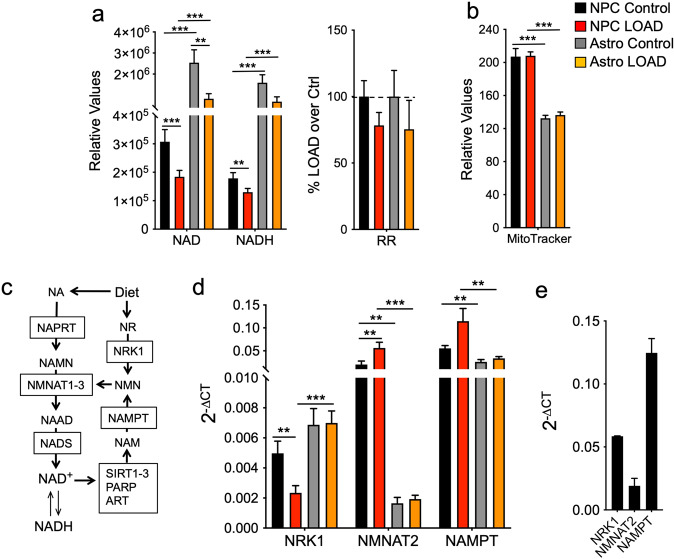
NAD^+^ synthesis and recycling is reduced in LOAD NPCs and astrocytes. **a** NAD^+^ and NADH levels and redox ratios (RR) of LOAD (*n* = 9, red bars) or Control (*n* = 5, black bars) NPCs, and LOAD (*n* = 9, orange bars) or Control (*n* = 5, gray bars) astrocytes. **b** Results from MitoTracker assays. **c** Schematic of NAD^+^ de novo synthesis or recycling. Dietary products, including nicotinic acid (NA) and nicotinamide riboside (NR), are converted to nicotinic acid mononucleotide (NAMN) or nicotinamide mononucleotide (NMN) by nicotinic acid phosphoribosyltransferase (NAPRT) or nicotinamide riboside kinase 1 (NMRK1 or NRK1), respectively. NAMN and NMN are substrates for nicotinamide mononucleotide transferases subtypes 1-3 (NMNAT1-3) to produce the NAD^+^ precursor nicotinic acid adenine dinucleotide (NAAD) that is converted to NAD^+^ by NAD synthase (NADS) [50, 51]. NAD^+^ is a co-substrate for SIRTs, PARPs, and ARTs and consumed to nicotinamide (NAM) that is recycled to NMN by nicotinamide phosphotransferase (NAMPT). **d**, **e** qRT-PCR results of NRK1, NMNAT2, and NAMPT in LOAD and Control NPCs and astrocytes (**d**), and in secondary human astrocytes (**e**), plotted as 2^-ΔCT^ values. Data are mean ± SEM from three to four repeat experiments. *p < 0.1; **p < 0.05; ***p < 0.01 using one-way ANOVA.

NAD^+^ is a key electron transfer molecule in mitochondrial redox metabolism through its reduction to NADH, and a required co-substrate for crucial enzymes in numerous cellular functions, including adenosine diphosphate (ADP)-ribose transferases (ARTs), poly(ADP-ribose) polymerases (PARPs), sirtuins (SIRTs), and cyclic ADP-ribose synthases (CD38 and CD157) [28, 29]. NAD levels and biochemical factors in its synthesis and/or recycling pathway diminish with age or in neurodegenerative diseases, including LOAD [28, 30, 31]. Consistent with those observations, we previously found that both NAD^+^ and NADH levels were substantially decreased, while the redox ratios (RR, NAD^+^/NADH) were slightly increased in LOAD skin cells [19]. When we assessed NAD^+^ and NADH in NPCs and astrocytes, levels of both metabolites were higher in astrocytes than in NPCs and reduced in LOAD NPCs and astrocytes versus Control lines (Fig. 2a). In addition, NAD^+^ and NADH showed strong positive correlations in all cells but there was a trend toward reduced RR in both LOAD NPCs and astrocytes (Fig. 2a and Supplementary Fig. 5), indicating that despite low levels of NAD^+^ and slightly lower RR, the general biochemical reducing power in LOAD is not compromised. We also showed that these reductions were not associated with diminished mitochondrial mass, as determined by MitoTracker assays that also showed that both LOAD and Control astrocytes had significantly less mitochondrial mass than NPCs (Fig. 2b). NAD^+^ is newly synthesized through dietary sources or recycled after consumption by SIRTs, ARTs, and PARPs that generate nicotinamide (NAM) as a side product which enters the NAD^+^ salvage pathway [28, 29] (Fig. 2c). To explore whether diminished NAD^+^ was a consequence of reduced de novo synthesis or recycling, we determined the gene expression of three key enzymes in the NAD^+^ pathway, nicotinamide mononucleotide transferase 2 (NMNAT2), nicotinamide phosphotransferase (NAMPT), and nicotinamide riboside kinase 1 (NRK1) (Fig. 2d). We found significantly more expression of NMNAT2 (12- to 30-fold) and NAMPT (2- to 3.5-fold) in NPCs compared to astrocytes. In NPCs, there was a reduction of NRK1 and increased expression of NMNAT2 and NAMPT in LOAD cells, while no differences were observed between LOAD and Control astrocytes. Finally, we detected similar gene expression patterns in secondary human astrocytes as observed in the iPSC-derived astrocytes (Fig. 2e). Together, these data demonstrate that reduced NAD^+^ and NADH levels in LOAD cells were associated with altered gene expression profiles of key factors in the NAD pathway in NPCs but not in astrocytes.

### Glucose uptake and IGF-1 or insulin responses are reduced in LOAD NPCs and astrocytes

**Fig. 3:**
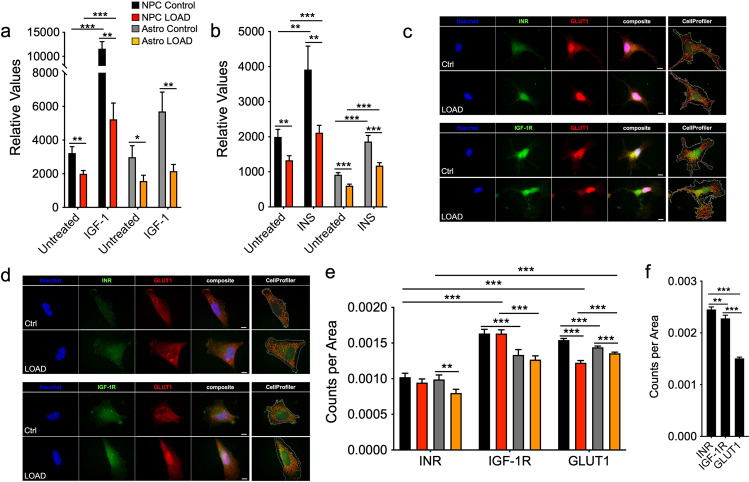
Glucose uptake and IGF-1 or INS responses are reduced in LOAD NPCs and astrocytes. **a**, **b** Glucose uptake in response to 100 ng/ml IGF-1 (**a**) or 400 mM INS (**b**) in LOAD (*n* = 8, red bars) and Control (*n* = 5, black bars) NPCs or LOAD (*n* = 9, orange bars) and Control (*n* = 5, gray bars) astrocytes. **c**, **d** Immunocytochemistry for INR (green), IGF-1R (green), and GLUT1 (red) on NPCs (**c**) and astrocytes (**d**). Nuclei are counterstained with Hoechst and CellProfiler overlay images are shown. Size bars = 20 µM. **e**, **f** Quantification of INR, IGF-1R, and GLUT1 punctae using CellProfiler software in LOAD (*n* = 8, red bars) and Control (*n* = 5, black bars) NPCs or LOAD (*n* = 7, orange bars) and Control (*n* = 5, gray bars) astrocytes (**e**) and human secondary astrocytes (**f**) plotted as normalized counts per cell area from *n* = 10 individual cells per cell line. Data are mean ± SEM from two repeat experiments. *p < 0.1; **p < 0.05; ***p < 0.01 using one-way ANOVA.

Glucose dysregulation and impairment or resistance to INS/IGF-1 signaling have been associated with both aging and LOAD [8, 32], and we previously observed that old Control and LOAD skin fibroblasts had an impairment of glucose uptake in response to IGF-1 [19]. Glucose uptake and the effects of IGF-1 or insulin (INS) in NPCs and astrocytes showed that astrocytes had overall lower glucose uptake than NPCs and both LOAD NPCs and astrocytes took up less glucose than Control cells (Fig. 3a, b). Treatment with IGF-1 or INS increased glucose uptake in all cells and, except for IGF-1-treated astrocytes, the response rates in LOAD cells were significantly lower than in Controls.

To identify mechanisms involved in reduced glucose uptake in LOAD cells, we determined INS receptor (INR), IGF-1 receptor (IGF-1R), and glucose transporter 1 (GLUT1) densities. ICC data showed typical punctae staining (Fig. 3c, d and Supplementary Fig. 6a–d), which we quantified using CellProfiler software (Fig. 3e). The analyses revealed that Control and LOAD NPCs had lower INR than IGF-1R and GLUT1 densities. While LOAD and Control NPCs exhibited similar IGF-1R densities, LOAD NPCs had reduced GLUT1. Astrocytes had significantly less INR than IGF-1R or GLUT1. There were also significantly fewer IGF-1R densities in astrocytes than in NPCs, while GLUT1 was lower in Control cells but higher in LOAD cells. Furthermore, both INR and GLUT1 numbers were reduced in LOAD astrocytes. INR, IGF-1R, and GLUT1 densities on secondary human astrocytes showed expression of all molecules, however, with different distributions than seen in the iPSC-derived astrocytes (Fig. 3f and Supplementary Fig. 6e). Finally, we performed correlation analyses on glucose uptake with INS or IGF-1. There were strong positive correlations for Control and LOAD NPCs or astrocytes treated with INS or IGF-1, respectively, and also for Control astrocytes treated with INS (Supplementary Fig. 7a–d). In contrast, Control and LOAD NPCs treated with IGF-1 and LOAD astrocytes treated with INS had weak positive or even negative correlations (Supplementary Fig. 7b, c).

These data demonstrate that LOAD NPCs and astrocytes exhibit lower glucose uptake in general and also reduced response rates to INS or IGF-1, which may be a consequence of lower cytoplasmic GLUT1 and/or INR but not altered IGF-1R densities.

### Processing of bioenergetic metabolites is altered in LOAD NPCs and astrocytes

To gain deeper insight into the molecular aspects of the bioenergetic and metabolic states of NPCs and astrocytes on a functional level, we performed Biolog MitoPlate S-1 assays, which assess mitochondrial function by measuring the rates of electron flow into and through the electron transport chain (ETC) from metabolic substrates that are processed in different bioenergetic pathways. Kinetic analysis of 31 substrates revealed cell-specific profiles for HEK293 cells (used as assay control), NPCs, and astrocytes, with NPCs exhibiting lesser metabolic activities than HEK293 cells and astrocytes, and iPSC-differentiated astrocytes showing bioenergetic characteristics similar to secondary human astrocytes (Supplementary Fig. 8).

**Fig. 4:**
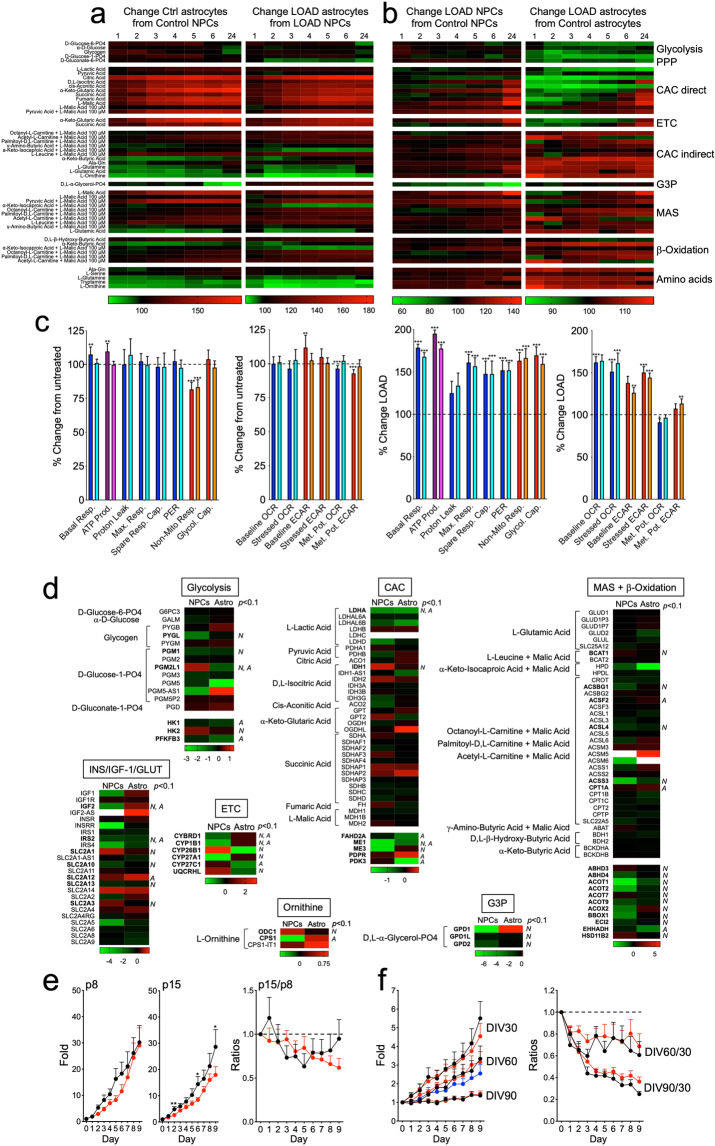
Processing of bioenergetic metabolites, their associated transcriptional profiles, and cell proliferation rates are all altered in LOAD NPCs and astrocytes. **a**, **b** Heat maps of data from Biolog experiments on LOAD (*n* = 9) and Control (*n* = 5) NPCs or LOAD (*n* = 9) and Control (*n* = 5) astrocytes for kinetic measurements at 1–6 and 24 h. Shown are percent changes in astrocytes versus NPCs (**a**) and LOAD versus Control cells (**b**) of 31 metabolites organized by association with metabolic function. Red indicates percent increase and green percent decrease. Data are mean ± SEM from triplicate measurements in two repeat experiments. **c** Treatment of Control (*n* = 5) and LOAD (*n* = 6) astrocytes with 3 mM BHB. Seahorse data are plotted as substrate effect, i.e., percent change in BHB-treated versus untreated cells (dark colors depict Control and light colors LOAD astrocytes) or as LOAD effect, i.e., percent change in LOAD versus Controls (untreated cells are in dark and BHB-treated cells in light colors). **d** Heat maps showing DEGs of factors involved in metabolizing bioenergetic substrates in glycolysis, CAC, MAS and β-oxidation, ETC, G3P shuttle, and L-ornithine metabolism, and DEGs related to INS/IGF-1 signaling and glucose uptake. RNA-Seq data are from *n* = 5 Control and *n* = 5 LOAD NPC and astrocyte samples (total *n* = 20). Terms in bold have significant changes with *p* values < 0.1. N NPCs, A astrocytes. **e**, **f** Data from cell proliferation assays measured for Control (black, *n* = 5) and LOAD (red, *n* = 8) NPCs (**e**) and Control (black, *n* = 5) and LOAD (red, *n* = 8) astrocytes, and human astrocytes (blue) (**f**). Data are plotted as fold change of cell numbers for 9 consecutive days relative to day 0 at NPC passages (p) 8 and 15, and astrocytes at DIV30, 60, and 90, or as ratios p15 over p8, or DIV60 or DIV90 over DIV30. Growth rates of secondary human astrocytes are plotted as average values for multiple measurements at passages p5 to p15. Data are mean ± SEM from duplicate or triplicate measurements in two repeat experiments. *p < 0.1; **p < 0.05; ***p < 0.01 using one-way ANOVA.

When we compared astrocytes with NPCs we found that both Control and LOAD astrocytes had higher metabolic rates of mitochondrial substrates directly linked to the CAC or ETC, the malate-aspartate shuttle (MAS), and β-oxidation, and had reduced metabolism of α-ketobutyric and L-glutamic acid, L-ornithine, L-glutamine, and tryptamine, than Control or LOAD NPCs (Fig. 4a and Supplementary Fig. 9). Interestingly, Control astrocytes also showed some increase in metabolizing glycolytic substrates compared to NPCs, which was less pronounced in LOAD astrocytes. There were also differences related to D,L-α-glycerol-phosphate (glycerol-3-phosphate (G3P)), a substrate in lipid synthesis and the glycerophosphate shuttle that together with MAS is an essential mechanism to transport reducing equivalents from cytosolic NADH to ETC [33]. Our data show that compared to NPCs, Control astrocytes had a stark decrease in D,L-α-glycerol-phosphate metabolism, while its metabolism was slightly increased in LOAD astrocytes.

When we compared LOAD with Control cells, in NPCs, glycolytic substrates, including α-D-glucose, D-glucose-6-phosphate, D-gluconate-6-phosphate, and glycogen, which act on the upstream events of glycolysis or PPP, induced slightly lesser electron flows in LOAD cells than in controls (Fig. 4b and Supplementary Fig. 9). As for substrates directly or indirectly activating or entering the CAC, ETC, or β-oxidation, some showed lower while others had higher metabolic rates. Specifically, L-lactic, pyruvic, citric, succinic, and cis-aconitic acid caused less electron transfer, while D,L-isocitric, α-ketoglutaric, fumaric, α-keto- and β-hydroxybutyric acid, or malic acid alone or in combination with pyruvic, γ-amino-butyric, α-keto-isocaproic acid, the carbon donor L-leucine, or the mitochondrial fatty acid transporters acetyl-, octanoyl-, or palmitoyl-carnitine, increased electron flow. L-ornithine, alanine-glutamine, L-glutamine, L-serine, and tryptamine that are amino acid substrates in other pathways linked to the mitochondrial respiratory chain also led to increased electron flow in LOAD cells compared to Controls. Most striking was a substantially lesser electron transfer in LOAD NPCs after application of D,L-α-glycerol-phosphate. The profiles in astrocytes demonstrated a stark reduction of the metabolism of the glycolytic substrates and those that are directly linked to the CAC and ETC, while processing of amino acids and those metabolites that are associated with MAS or indirectly activate the CAC and are related to fatty acid β-oxidation was increased.

The Biolog experiments showed that the ketone body β-hydroxybutyrate (BHB), which has been suggested to improve brain energy metabolism and dementia symptoms in LOAD patients [34–37], had only a small effect in boosting β-oxidation in either Control or LOAD NPCs or astrocytes. To evaluate this finding in more detail, we treated Control and LOAD astrocytes with BHB and performed Seahorse Mito Stress Tests. We first evaluated a dose effect of BHB on secondary human astrocytes, observing a small increase of respiratory activity with 3 and 6 mM BHB, a larger increase with 12 mM, and a stark decrease of bioenergetic functions with 18 mM BHB (Supplementary Fig. 10a). These effects were accompanied by a slight increase of CyQuant fluorescence at 3 and 6 mM BHB, and a stark decrease at 12 and 18 mM BHB, suggesting decreased cell viability at high BHB doses (Supplementary Fig. 10b). We next tested Control and LOAD astrocytes with 3 and 12 mM BHB and, consistent with the Biolog data, there was no major treatment effect with 3 mM BHB in either Control or LOAD cells, though there was a small increase in basal respiration and ATP production in the control cell lines (Fig. 4c). Also, BHB did not change the observed profile of increased bioenergetic functions in LOAD astrocytes. Treatment with 12 mM BHB revealed a decrease of respiratory and glycolytic functions both in Control and LOAD astrocytes, apparently causing cell stress, as cell viability was reduced (Supplementary Fig. 10c, d).

Together, these findings demonstrate a bioenergetic and metabolic shift of developing astrocytes toward increased mitochondrial respiration (CAC, ETC, MAS, and β-oxidation) but with a reduction in metabolizing amino acids. In addition, the data point to differences in LOAD cells with regard to the production or transfer of reducing agents into the mitochondria and alterations at the interphase of glycolysis and the mitochondrial respiratory chain. LOAD cells have deficiencies in activating CAC, ETC, and glycolysis but increased β-oxidation, amino acid metabolism, and MAS. Finally, treatment with the ketone body BHB did not markedly increase bioenergetic functions or change any LOAD-associated bioenergetic phenotypes.

### LOAD and Control NPCs and astrocytes exhibit differences in their metabolic transcriptomes

To determine if the observed changes in bioenergetic functions in LOAD cells are consequences of altered gene expression profiles, we analyzed the transcriptomes of *n* = 5 Control and *n* = 5 LOAD NPCs and astrocytes (total *n* = 20 samples) using RNA-Seq. Unbiased hierarchical clustering revealed robust gene expression profiles distinctive for Control or LOAD cells (Supplementary Fig. 11a) and a number of differentially expressed genes (DEGs). We focused our analysis on those genes encoding enzymes that are involved in the pathways that process bioenergetic substrates analyzed in the Biolog assays (Fig. 4d and Supplementary Tables 2 and 3). In glycolysis, significant changes were found for glycogen phosphorylase (*PYGL*), phosphoglucomutase (*PGM1*, *PGM2L1*), hexokinase (*HK1* and *2*), and phosphofructokinase (*PFKFB3*, *PFKP*), and consistent with the reduction in glycolysis observed in the Biolog assays there was a predominant downregulation of these genes in LOAD. We also found a significant downregulation of lactate dehydrogenase A (*LDHA*), which converts lactate to pyruvate as the major substrate to fuel the respiratory chain, in LOAD NPCs and astrocytes. In contrast to a stark reduction of CAC activity in LOAD astrocytes (see Fig. 4b), other than isocitrate dehydrogenase 1 (*IDH1*), none of the enzymes that directly act in the CAC were significantly differentially expressed between LOAD and Control cells. However, there was significant downregulation of other CAC-associated genes, such as fumarylacetoacetate hydrolase domain containing 2A (*FAHD2A*), malate enzyme (*ME1* and *2*), pyruvate dehydrogenase phosphatase regulatory subunit (*PDPR*), and pyruvate dehydrogenase kinase (*PDK3*). Similar observations were made for MAS- and β-oxidation-related enzymes, where there were several downregulated genes predominantly in LOAD NPCs but no obvious association with the functional Biolog data. α-ketoglutaric and succinic acid are substrates for both CAC and ETC enzymes: glutamic-pyruvic transaminase (*GPT*) and OGDH in the CAC, as well as complex I in the ETC use α-ketoglutaric acid as substrate, and succinic acid dehydrogenase (*SDH*), which comprises complex II and also acts in the CAC, metabolizes succinic acid. Both substrates are less metabolized in LOAD astrocytes but not in NPCs. However, neither *SDH*, or *GPT*, *OGDH*, nor the NADH:ubiquinone oxidoreductase (*NDUF*) subunits of complex I were differentially expressed in LOAD cells, indicating no direct association between gene expression and functional outcome of the actions of α-ketoglutaric and succinic acid. Interestingly, in the ETC, only subunits of cytochrome p450 that are involved in fatty acid and cholesterol oxidation were significantly dysregulated in LOAD. With regard to the diminished metabolism of G3P in LOAD NPCs and the increased metabolism of L-ornithine in LOAD astrocytes or NPCs, we found significant downregulation of *GPD1*, *GPDHL*, and *GPDH2* in LOAD NPCs, and upregulation of the L-ornithine processing enzymes ornithine decarboxylase 1 (*ODC1*), and carbamoylphosphate synthetase (*CPS1*, *CPS1-IT1*) in both LOAD NPCs and astrocytes. We also assessed gene expression of elements in the INS/IGF-1 signaling cascades and members of the solute carrier family 2 (*SLC2*) encoding GLUT proteins. We found no indication that the genes encoding *INR*, *IGF-1R*, or *GLUT1* were differentially expressed between LOAD and Control cells; however, several genes in the signaling pathways were downregulated in LOAD NPCs and astrocytes, including *IGF-1* and *2*, insulin receptor related receptor (*INSRR*), and insulin receptor substrate 2 (*IRS2*).

To substantiate some of the RNA-Seq data in more detail, we performed additional quantitative reverse transcriptase polymerase chain reaction (qRT-PCR) experiments including the cytosolic and mitochondrial forms of GPDH (*GPDH1* and *2*, respectively), and the CAC enzymes *IDH3A*, oxoglutarate dehydrogenase (*OGDH*), and cytosolic or mitochondrial malate dehydrogenase (*MDH1* or *2*, respectively) (Supplementary Fig. 11b, c). In NPCs, the expression of *GPDH1* was 50- to 500-fold lower than those of the other enzymes and was below the detection threshold in astrocytes. *GPDH1* and *2*, *MDH1*, *IDH3A*, and *OGDH* were expressed less, while *MDH2* was expressed more in LOAD NPCs. In LOAD astrocytes, *GPDH2* and *IDH3A* expression was also reduced, while *MDH2* was increased and *MDH1* and *OGDH* expression was unchanged. For the mitochondrial forms of *GPDH* and *MDH* versus the cytosolic forms, *GPDH2* was 4,560% ± 1,432% (*p* = 0.006) and 20,290% ± 3,366% (*p* ≤ 0.00001) more expressed in Control or LOAD NPCs than *GPDH1* and, respectively, *MDH2* was 37.2% ± 6.2% (*p* = 0.003) lower and 136.7% ± 14.5% (*p* = 0.004) higher expressed than *MDH1*.

### LOAD and Control NPCs but not astrocytes exhibit changes in cell proliferation rates

To assess if the observed bioenergetic and metabolic changes in LOAD cells influenced cell growth, we determined the proliferation rates for NPCs and astrocytes (Fig. 4e, f). In LOAD NPCs there was a slight reduction of growth rates in late compared to early passages as well as a tendency to slower growth rates at later days (day 7–9) in culture compared to Control cells (Fig. 4e). In contrast, both LOAD and Control astrocytes had continuous reductions in growth rates in vitro at days (DIV)60 and DIV90 compared to DIV30, and growth rates similar to secondary human astrocytes at DIV > 60 (Fig. 4f). These data indicate that bioenergetic changes in LOAD may be associated with modestly reduced proliferation characteristics of NPCs, but not astrocytes, as the observed reduction in cell growth appears to be an effect related to the differentiated astrocytic cell type, rather than to their iPSC origin.

## Discussion

**Fig. 5:**
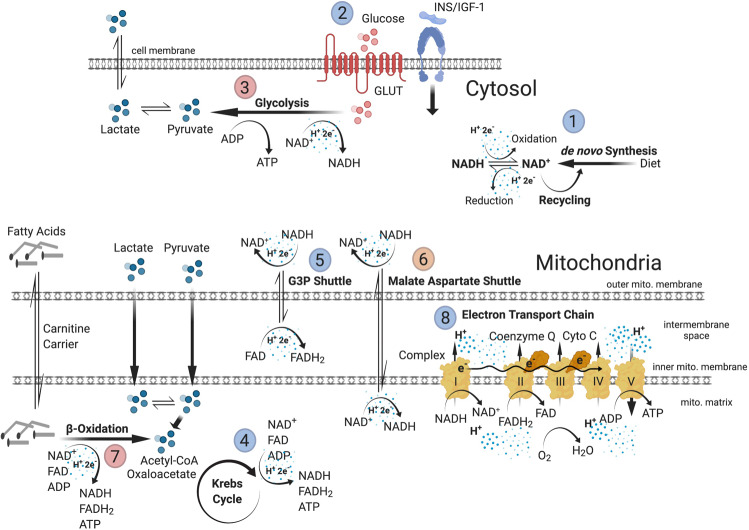
Bioenergetic and metabolic alterations in LOAD. Summary of energy flow and substrate metabolism at bioenergetic “branch points” where changes in LOAD cells have been identified in this study. Upregulated processes are indicated in red and downregulated processes in blue. Production of NAD and NADH to transfer protons (H^+^) and electrons (e^–^) is reduced in LOAD cells (1). Glucose uptake and INS/IGF-1 signaling (2) are impaired and baseline glycolytic activities (3) are increased in LOAD cells; however, stimulation of glycolysis by glycolytic substrates is diminished. In addition, LOAD cells have reduced metabolism of lactate, a key metabolite produced by astrocytes and released for uptake by neurons to support energy production [13, 20, 22]. Lactate is converted from or can be converted to pyruvate that is the end product of glycolysis and metabolized in the mitochondria to Acetyl-CoA and oxaloacetate to fuel the Krebs cycle (4) that generates the reducing agents FADH_2_ and NADH, and ATP. LOAD cells have deficiencies in activating the Krebs cycle. Because glycolytically produced cytosolic NADH cannot pass the outer mitochondrial membrane, protons and electrons are transported through the G3P shuttle (5) and MAS (6) that generate FADH_2_ in the intermembrane space and NADH in the mitochondrial matrix, respectively. LOAD NPCs have diminished G3P shuttle activity and both LOAD NPCs and astrocytes exhibit increased activation of MAS. LOAD cells also have increased transport of fatty acids to the mitochondria through carnitine carriers and their metabolism in β-oxidation (7), as an alternative pathway to produce FADH_2_, NADH, ATP, and Acetyl-CoA. The protons and electrons carried by NADH and FADH_2_ are used to generate a proton gradient in the ETC (8), which includes complexes I-V, coenzyme Q, and cytochrome C, and leads to the production of ATP by OxPhos. In general, OxPhos is increased in LOAD cells; however, LOAD astrocytes have diminished direct substrate activation of complex I and II. Finally, the functional changes at these bioenergetic and metabolic “branch points” are associated with transcriptional changes of genes that are involved in these processes.

LOAD is a complexly determined disorder characterized by a combination of multiple interacting pathological processes, some of which are genetically determined and inherent in neurodevelopment and in youth and some of which are part of the normal aging process. Among these key factors are changes in bioenergetics and metabolism [3–9, 14–18]. Abnormalities of glucose processing and the balance between glycolytic and mitochondrial energy production have been documented in LOAD; however, the degree to which these anomalies are inherent or acquired has not been previously established. Here, we demonstrate that NPCs and astrocytes as cell populations essential in early CNS development and as key players in mature brain metabolism have numerous bioenergetic and metabolic changes and deficits associated with LOAD (Fig. 5).

We found that compared to NPCs astrocytes had higher respiratory and glycolytic activities and NAD/NADH levels but less mitochondrial mass, amino acid metabolism, INS-mediated glucose uptake, and IGF-1 and GLUT1 densities than NPCs, demonstrating the acquisition of cell-specific metabolic phenotypes during development. Independent of these characteristics, LOAD cells exhibit shifts in bioenergetic and metabolic functions including low levels of NAD, diminished capacity to uptake glucose, and deficits in the production and transfer of reducing agents in the glycolytic process and the mitochondrial respiratory chain, with the cells seeming to overcompensate by upregulating OxPhos and glycolysis. In addition, LOAD NPCs had substantial reductions of the spare respiratory capacity indicating diminished respiratory plasticity. While the molecular causes of reduced NAD synthesis need further investigation, our results demonstrate that reduction in glucose uptake and response rates to INS and IGF-1 in LOAD could be a consequence of lower INR and GLUT1 receptor densities and/or altered expression of factors in INS/IGF-1 signaling pathways. Glucose dysregulation with elevated glucose levels and decreased expression of glucose transporters and enzymes in the glycolytic pathway have been observed in AD patients’ brains [38], and impairment or resistance to INS/IGF-1 signaling has long been associated with aging and the development of LOAD [8, 32]. The molecular mechanisms have been, amongst others, attributed to dysfunctions in the INS/IGF-1 signaling cascade [39, 40], which is supported in our study by the observed transcriptional downregulation of factors, such as IRS2 and 4 in LOAD NPCs and astrocytes, and IGF-1 and 2, and INSRR predominantly in NPCs. Importantly, our data show that altered glucose uptake and INS/IGF-1 signaling already occur in early development and, thus, could be a general and an inherent aspect in cells of individuals that develop INS/IGF-1 resistance as a potential risk for LOAD later in life.

We found a striking reduction of D,L-α-glycerol-phosphate metabolism in the development of Control astrocytes and in LOAD NPCs, indicating diminished GPDH and G3P shuttle activity to transport reducing equivalents from cytosolic NADH to the ETC [33]. Mammalian astrocytes express GPDH during development and postnatally [41–43], and rather than using MAS, seem to mostly rely on the G3P shuttle, which can be activated by glucose and lactate oxidation (reviewed in McKenna et al. [43]). In contrast, using the iPSC paradigm, our data show that differentiating human astrocytes seem to shift from G3P to MAS activity. Overall, there were only a few bioenergetic differences between developing Control or LOAD astrocytes; however, there were substantial changes in the bioenergetic activities of LOAD versus Control cells. These changes mainly pertained to a reduction of the glycolytic, CAC, and ETC capacities, and increases in β-oxidation and MAS, which are more pronounced in LOAD astrocytes. Overall, the striking decrease in G3P shuttle and the increase in MAS and ETC activity in LOAD NPCs, and the increased metabolism of amino acids (alanine-glutamine, L-glutamine, and tryptamine, but not L-serine) and L-ornithine, which predominantly occurs in LOAD astrocytes, suggest that LOAD cells have major deficits in multiple bioenergetic pathways, including glycolysis, CAC, ETC, and G3P shuttle, for which they seem to compensate by boosting other pathways, notably MAS and β-oxidation, and amino acid metabolism (Fig. 5). We also determined that the bioenergetic profiles in astrocytes were not changed by treatment with physiological concentrations (up to 6 mM) of BHB, suggesting a limited effect of ketone bodies on altering cellular energy management in these cells. It should be noted, however, that a BHB effect could have been masked, as these experiments were not performed under conditions of oxidative stress, starvation, or glucose blockage, which have been associated with shifting energy production to BHB-activated β-oxidation [44].

The LOAD-associated metabolic alterations observed here could, in part, be the consequences of DEGs. LOAD NPCs and astrocytes exhibit transcriptional deregulation of several molecules in key functions and branch points in bioenergetics processes, such as *PGM*, *HK*, and *PFKFB3*, which regulate upstream events in glycolysis; *LDHA* that controls the production of pyruvate; *ME* that works together with *LDH* catalyzing the conversion of malate to pyruvate and is essential for *NADPH* regeneration; *PDPR* and *PDK3*, which are important in oxidative decarboxylation of pyruvate; and *GPDH* as the determining factor in the G3P shuttle. In addition, the lack of CAC and complex I- to V-associated DEGs and the predominance of DEGs related to cytochrome p450 and β-oxidation indicate that the transcriptional deregulation in LOAD affects only certain aspects of the respiratory chain. The exact consequences of these LOAD-associated bioenergetic phenotypes need further investigation; however, to this end, we could show that growth of LOAD NPCs slowed-down after multiple passaging, while both LOAD and Control astrocytes had similar growth patterns. The diminished growth rate of LOAD NPCs is consistent with data from a recent study by Meyer et al. [45], who observed reduced NPC proliferation in combination with accelerated neurogenesis in cells derived from sporadic AD iPSC when compared to normal controls.

In sum, LOAD cells have deficits in efficiently producing energy due to impairments in numerous key components of bioenergetic substrate uptake or production, while they seem to enhance alternative pathways for energy production and transport. Overall, it appears that LOAD cells “work harder” to produce and maintain energy balance in both baseline conditions and under stress. Importantly, these changes are inherent, probably body-wide, as also observed in fibroblasts [19], and already occur in early development, all suggesting that individuals with such altered bioenergetic and metabolic features may be predisposed to the risk and pathophysiology of LOAD. LOAD may, thus, be a consequence of a lifelong altered and inefficient energy management leading to progressive homeostatic imbalances and associated downstream effects including the inability to sufficiently compensate for neurotoxic insults, all contributing to an aberrant aging process resulting in neural dysfunction and degeneration at the cellular level, and dementia at the cognitive level [9, 10, 30, 46–48]. The new results presented here provide important details characterizing differences in energy metabolism inherent to cells in those at risk for LOAD. While developing interventions for multi-hit disorders like LOAD is complicated, the findings presented here offer a platform that could be used to better define and address LOAD-associated pathophysiology. Further study of these abnormalities might determine control points and modulators to normalize bioenergetic functions, aid in developing tests for risk, and could lead to effective preventive measures in LOAD.

## Methods

### Subject population and cell derivation

Subjects (Supplementary Table 1) were recruited at the McLean Hospital Memory Diagnostic Clinic. PBMCs were derived from blood samples and dermal fibroblasts from skin biopsies as described [25]. All samples were genotyped for APOE. Fibroblasts or PBMCs were reprogrammed to iPSC using the Sendai virus methodology, except for two published lines (C1 and C2 [25, 26]), which were converted by RNA reprogramming. All iPSC lines were rigorously characterized for pluripotency.

### Cell culture

Cells were differentiated and propagated in standard tissue culture. iPSCs were dissociated to single cells to form embryonic bodies from which NPCs were derived. Astrocytes were differentiated from NPCs according to published protocols [49]. Cell phenotypes were determined by ICC and cell proliferation was measured with a Scepter^TM^ cell counter (MilliporeSigma, Burlington, MA). Cells stained by ICC were visualized using an Axiovert 200 M inverted microscope (Carl Zeiss, Inc.) and captured with the Zeiss Axiocam 503 Mono and Zeiss Zen Lite program. To quantify cell surface molecules, CellProfiler version 3.1.8. software was used (www.cellprofiler.org).

### Biochemical assays

Bioenergetic parameters were determined using Seahorse XFp Cell Mito Stress Tests (Seahorse, Agilent Technologies, Santa Clara, CA) and the processing of bioenergetic substrates were assessed in Biolog MitoPlate S-1 assays (Biolog, Hayward, CA). To determine biochemical compounds and function, the NAD/NADH-Glo™ and the Glucose Uptake-Glo™ Assay Kits (Promega, Madison, WI) were used. Mitochondrial densities were determined with the MitoTracker^TM^ Green FM dye (Invitrogen Thermo Fisher Scientific, Waltham, MA).

### Molecular assays

Total RNA was isolated and purified using TRI-Reagent (MilliporeSigma) and RNA integrity was measured with an Agilent Bioanalyzer 2100 (Agilent). RNA was used for qRT-PCR and in RNA-Seq performed on an Illumina NovaSeq 6000 system.

### Statistical analysis

One-way analysis of variance tests for independent measures were performed using the Social Science Statistics software (http://www.socscistatistics.com/Default.aspx) or PRISM 8 for macOS Version 8.1.0. Differences of comparison were considered statistically significant when *p* values were less than 0.05, while *p* values between 0.05 and 0.1 were considered trend data.

Detailed information of the materials and methods is presented in the Supplement Information.

## Supplementary information

Supplemental Material

Table S2
