# Supplementary material for: Brain cells derived from Alzheimer’s disease patients have multiple specific innate abnormalities in energy metabolism
Source: Mol Psychiatry. 2021 Apr 16;26(10):5702–14. doi: 10.1038/s41380-021-01068-3 (PMC8758493; doi:10.1038/s41380-021-01068-3)
Supplement: Supplementary file 1 — Supplemental Material [file 41380_2021_1068_MOESM1_ESM.pdf]

Brain cells derived from Alzheimer's disease patients have specific innate abnormalities in energy metabolism

Woo-In Ryu, PhD[1,2,3], Mariana K. Bormann, MS[1,2,3], Minqi Shen, MS[1,2,3], Dohoon Kim, PhD[4], Brent Forester, MD, MS[1,5], Yeongwon Park, MS[6], Jisun So, PhD[7], Hyemyung Seo, PhD[6], Kai-C. Sonntag, MD, PhD[1,2,3,8]*, Bruce M. Cohen, MD, PhD[1,3]*

[1]Department of Psychiatry, [2]Basic Neuroscience Division, [3]Program for Neuropsychiatric Research, McLean Hospital, Harvard Medical School, Belmont, MA 02478
[4]Department of Immunology, Tufts University School of Medicine, Boston, MA 02111
[5]Mood Disorders Division and Geriatric Psychiatry Research Program, McLean Hospital, Harvard Medical School, Belmont, MA 02478
[6]Department of Molecular and Life Sciences, Hanyang University, 55 Hanyangdaehakro-, Sa-dong-dong, Sangrok-gu, Ansan, South Korea
[7]Jean Mayer USDA Human Nutrition Research Center on Aging, Tufts University, Boston, MA 02111

Supplement Information

Methods

Subject population

Subjects (Supplementary Table 1) were recruited at the McLean Hospital Memory Diagnostic Clinic and diagnosed by a geriatric psychiatrist using the Diagnostic and Statistical Manual of Mental Disorders (DSM-IV) criteria and the Montreal Cognitive Assessment (MOCA) score. Cell samples from nine LOAD patients and five non-demented control subjects were used in the current study.

Derivation of dermal fibroblasts and peripheral blood mononuclear cells

Fibroblasts were derived from skin biopsies as described [1] or purchased from ATCC (D551 and HFF1 cell lines) and cultured in MEM media (Thermo Fisher Scientific, Waltham, MA) supplemented with 15 % FBS, 100 U/ml Penicillin/Streptomycin (Thermo Fisher Scientific) and 2 mM GlutaMAX™ (Invitrogen). The fibroblast cell lines C3, C5, AD2, AD3, AD4, AD5, and AD6 (Supplementary Table 1) were previously published in [2]. Peripheral blood mononuclear cells (PBCS) were prepared from blood samples drawn into BD Vacutainer[a] Glass Mononuclear Cell Preparation (CPT) Tubes Heparin 8 ml (Thermo Fisher Scientific/Beckton Dickinson) by centrifugation and collection of buffy coats. Cells were stored in the cell bank of the Program for Neuropsychiatric Research at McLean Hospital. All experiments and procedures were performed in accordance with relevant guidelines and regulations and were approved by the Partners Health Care Institutional Review Board with written informed consent provided by all subjects.

Generation of iPSC lines and culture

Dermal fibroblasts or PBMCs were reprogrammed to iPSC at the New York Stem Cell Foundation Research Institute (NYSCF), at the University of Carolina Human Pluripotent Stem Cell Core Facility, or at the Tsai Laboratory Picower Institute for Learning and Memory (director, Dr. Li-Huei Tsai) (Supplementary Table 1). All lines were reprogrammed using the Sendai virus methodology, except for lines C1 and C2, which were converted by RNA reprogramming. These two lines were used in previously published studies [1, 3]. Newly generated iPSC lines were screened by immunocytochemistry for expression of the pluripotent stem cell markers OCT4, SOX2, TRA-60, and TRA-181, and the TaqMan hPSC scorecard assays (Thermo Fisher Scientific) (Supplementary Fig. 1). All lines were karyotyped at the core facilities or at Cell Line Genetics (Madison, WI) and expressed stable normal karyotypes. iPSC lines were given to the laboratory at passages 5-7 and were then expanded in feeder-free cultures on Vitronectin (VTN) (Gibco, MA, USA) coated plates in Essential 8™ Medium (Gibco) and propagated using ReLeSR (StemCell Technologies, Vancouver, BC, Canada).

Other cell lines

HEK293 human embryonic kidney cells were purchased from ATCC (#CRL-1573) and secondary human astrocytes isolated from cerebral cortex were obtained from ScienCell Research Laboratories (#1800) (ScienCell, Carlsbad, CA, USA). The cell lines were propagated in culture conditions recommended by the manufacturers.

Generation of NPCs and astrocytes

For differentiation, iPSCs were dissociated to single cells and plated in AggreWell™ culture dishes (StemCell Technologies, Vancouver, CA) to form embryonic bodies (EB) in Neural Induction Media (StemCell Technologies) supplemented with 2 μM dorsomorphin (Peprotech, NJ, USA), 10 μM SB431542 (Peprotech), and 0.2 μM LDN (Peprotech). At day *in vitro* (DIV) 5, EBs were transferred to vitronectin coated plates and cultured in the same media. Clusters of NPC-containing rosettes were observed after DIV 6 and selectively picked and replated on vitronectin-coated culture dishes. After growth to confluency, NPCs were plated at a density of 1-2 x 10^5 cells/cm^2 using Accutase™ (StemCell Technologies) and cultured in Neuro Basal Media (1:1 composition of Neurobasal A media (Gibco) and DMEM:F12 (Gibco)), supplemented with 1 X B27 supplement without Vitamin A (Gibco), 1 X N2 supplement (Gibco), 1 X NEAA (Invitrogen, CA), 2 mM GlutaMax (Invitrogen), 1 % Penicillin/Streptomycin (Invitrogen), 100 μM β - mercaptoethanol (Sigma-Aldrich, St. Louis, MO, USA), 10 ng/ml EGF (Peprotech), and 10 ng/ml bFGF (Peprotech). NPCs were passaged every 5-7 days using Accutase and characterized for expression of SOX1, PAX6, and NESTIN by immunocytochemistry (ICC) at passage 3.

To differentiate astrocytes, NPCs were plated at a density of 1 x 10^5 cells/cm^2 on vitronectin-coated plates and, the next day, media was changed to astrocyte media (ScienCell) supplemented with 10 ng/ml BMP-4, 10 ng/ml CNTF, and 10 ng/ml Heregulin-β1 (all Peprotech) according to published protocols [4]. Astrocytes were grown to confluency and propagated every 5-7 days at a seeding density of 1 x 10^4 cells/cm^2 using Accutase. The cells were characterized for expression of GFAP, EEAT1, and S100 β by ICC at DIV30.

Cell proliferation assay

At each passage, NPCs were plated in 24 wells coated with vitronectin at a density of 2 x 10^5 cells/well. Cells were dissociated with Accutase and consecutively counted for 9 days using a Scepter™ cell counter (MilliporeSigma, Burlington, MA).

Seahorse XFp Cell Mito Stress Test

Seahorse XFp Cell Mito Stress Tests (Seahorse, Agilent Technologies, Santa Clara, CA) were performed as previously described [2] on an XFp instrument. Briefly, two days prior assay, Seahorse culture plates were coated with vitronectin for 1 hr at 37 °C and stored at 4 °C. One day prior assaying, 20,000 NPCs and 10,000 astrocytes were plated and cultured in the respective media overnight or for 24 hours in the presence of different concentrations of β-hydroxybutyrate (Sigma Aldrich, 166898) as indicated. At the day of the assay, XF assay medium was supplemented with 10 mM glucose, 1 mM pyruvate, and 2 mM glutamine, and the pH was adjusted to 7.4. After assay performance, cells were stained with CyQuant solution (Life Technologies - Thermo Fisher Scientific, Carlsbad, CA) diluted in XF assay medium and incubated for 1 hr at 37 °C. Green fluorescence (excitation: 485/20, emission: 528/20) was measured using a Synergy HT BioTek plate reader (BioTek Instruments, Winooski, VT) and values used for data normalization. Data analysis was performed using the Seahorse XF^e Wave software, including the Seahorse XF Cell Energy Phenotype Test Report Generator. This algorithm determines the OCR and ECAR baseline phenotypes (measurement of the cells' relative utilization of mitochondrial respiration and glycolysis under starting conditions), the OCR and ECAR stressed phenotypes (measurement of the cells' relative utilization of mitochondrial respiration and glycolysis when stressed) and the respective metabolic potentials as percentage increase of stressed OCR or ECAR over baseline OCR or ECAR.

The Seahorse Mito Stress Tests determine the oxygen consumption rate (OCR, pmol/min) and the extracellular acidification rate (ECAR, mpH/min) after injection of specific pharmacologic stressors that target the electron transport chain (ETC) and ATP production: Oligomycin, which inhibits complex V (ATP synthase), decreasing OCR and ATP production; Carbonyl cyanite-4 (trifluoromethoxy) phenylhydrazone (FCCP), which disrupts the mitochondrial membrane potential and collapses the proton gradient at the ETC leading to maximal respiration (O_2 consumption by complex IV); and Rotenone/Antimycin A, which inhibit complex I and III resulting in the shutdown of mitochondrial respiration. From the data generated, several measures can be calculated, including the basal and maximal respiration, and the spare respiratory capacity (maximal respiration minus basal respiration), the proton leak, non-mitochondrial respiration, and the coupling effect, which determines ATP production relative to basal respiration. The ECAR from which the proton efflux rate (PER, pmol H+/min) can be calculated are indirect measures of the cells' glycolytic capacity.

NAD/NADH Assay

NAD/NADH-Glo™ Assay Kits (Promega, Madison, WI) were performed with 2 x 10^5 cells according to protocols provided by the manufacturer. Cells were lysed with 1 % DTAB in 0.2 N NaOH and half of the samples were prepared for measuring NAD^+ by adding 0.4 N HCl (Sigma) and heating at 60 °C for 15 min. After deactivating NADH in these samples, Trizma base (Sigma) was added to the acid-treated samples. The other half of the samples was prepared to measure NADH by heating at 60 °C for 15 min and adding HCl/Trizma solution. After the samples were prepared, luminescence was measured using a Synergy HT BioTek plate reader (BioTek).

Glucose uptake assay

Glucose uptake was performed using the Glucose Uptake-Glo™ Assay Kit (Promega). NPCs were plated at a density of 2 x 10^4/well and astrocytes at 1 x 10^4 cells/well in 96 well culture dishes. Cells were incubated without glucose for 45 min and treated with 100 μg/ml IGF-1 (Peprotech) or 400 nM insulin (Sigma) for 15

min. Cells were then treated with 1 mM 2-deoxyglucose (2DG) and incubated for 20 min at room temperature. Subsequently, Stop Buffer and Neutralization Buffer were added sequentially. Before reading the luminescence using a Synergy HT BioTek plate reader (BioTek), 2DG6P Detection Reagent was added and incubated for 1 hr at room temperature. After assay performance, proteins were measured using the Bio-Rad Protein Assay for data normalization.

MitoTracker
NPCs were plated at a density of 2 x 10^5 and astrocytes 1 x 10^5 cells in VTN-coated black 96 well culture dishes. The next day, cells were stained with MitoTracker™ Green FM (Invitrogen) and Hoechst and fluorescence was measured with 485 nm excitation and 520 nm emission using a Synergy HT BioTek plate reader (BioTek).

Biolog Assays
Bioenergetics substrate metabolism was measured using Biolog MitoPlate S-1 assays (Biolog, Hayward, CA) according to the manufacturer's instructions. The MitoPlates were preincubated with 70 µg/ml saponin (Sigma), Biolog Mitochondrial assay solution, and Redox dye for 1 hr at room temperature. After the incubation, 60,000 NPCs and 40,000 astrocytes per well were added to each plate and the OD 590 was measured at various time points (0, 1, 2, 3, 4, 5, 6, and 24 hrs) using a Synergy HT BioTek plate reader (BioTek). Measurements were normalized to and calculated as percent change from no substrate control.

APOE genotyping
DNA from cells was extracted and a portion of the APOE gene containing the polymorphic single nucleotides rs429358 and rs7412 amplified by PCR using the primers FW: 5'-CTGATGGACGAGACCATGAAG-3' and RV: 5'- GGCTCGAACCAGCTCTTGAG-3'. PCR reactions were according to the Platinum™ Taq DNA Polymerase protocol (Invitrogen, Thermo Fisher) in 25 µl volumes containing 1 U Platinum™ Taq DNA Polymerase, 0.2 µM of each primer, 8 % KB Extender and 50 ng genomic DNA. The PCR cycling conditions were as follows: Initial denaturation at 94 °C for 2 min followed by 35 cycles with denaturation at 94 °C for 30 sec, annealing at 55 °C for 50 sec, extension at 72 °C for 50 sec; then a final extension at 72 °C for 10 min. All PCR products were purified by ExoSAP-IT™ PCR Product Cleanup Reagent (Applied Biosystems) and sent for sequencing at the MGH Center for Computational and Integrative Biology (CCIB) DNA Core using the same primer pair.

RNA isolation
RNA samples were isolated from cultured cells using TRI-Reagent (Sigma) according to the manufacturer's instructions. Briefly, the cells were homogenized in 1 ml of TRI reagent and incubated for 5 min at room temperature (RT). Then, 200 µl of chloroform (Merck, Darmstadt, Germany) was added, vigorously mixed, and incubated for 15 min at RT. After the mixture was centrifuged at 12,000 g for 15 min at 4 °C, the separated aqueous phase was transferred to a new tube. To precipitate RNA, 500 µl of 2-propanol (Merck) per ml of TRI Reagent was added and inverted gently, incubated for 10 min at RT, and centrifuged at 12,000 g for 10 min at 4 °C. After centrifugation, the supernatant was removed, and the RNA pellet was washed with 1 ml of 75 % ethanol (Merck) per ml of TRI Reagent. The samples were centrifuged at 7,500 g for 5 min at 4 °C, then the supernatants removed again, and the RNA pellet was dried for 20 min. After drying, total RNA pellets were dissolved in nuclease-free water. RNA quality and quantity were assessed using Nano Drop 8000 spectrophotometer (Thermo Scientific) and total RNA integrity was checked using Agilent Bioanalyzer 2100 (Agilent) with an RNA Integrity Number (RIN) value.

Quantitative reverse transcriptase Polymerase Chain Reaction (qRT-PCR)
For qRT-PCR, 2-4 µg of total RNA was converted to cDNA using the High Capacity cDNA Reverse Transcription (Applied Biosystems – Thermo Fisher Scientific, Foster City, CA) in 20 µl per reactions following the protocol provided by the company. qPCR was conducted on a BioRad CFX Connect PCR cycler (BioRad) using SsoAdvanced SYBR Green Supermix (BioRad). The settings for the cycler were as follows: an initial incubation of 10 min 95 °C and subsequent repeats of 15 sec 95 °C, 15 sec 55 °C, 1 min 60 °C (50x). PCR data were analyzed using the $2^{-\Delta CT}$ method to normalize each target gene against β-actin (BACT).

The following primers were used:

Primer Name	Forward (5' -> 3')	Reverse (5' -> 3')	Reference
**BACT**	TCACCAACTGGGACGACATG	GTCACCGGAGTCCATCACGAT	[5]
**IDH3A**	TGCTGCCAAAGCACCTATTCA	GTGACCGGCTGCTATTGGG	[2]
**GPD1**	TGCTGAATGGGCAGAAAC	AAATGTGGTGGCATGAGG	[6]
**GPD2**	CGGACAACATAACGATGCAC	CTGTCTGGGGGTCTGTCTTC	[7]
**MDH1**	CATCCCCAAGGAGAACTTCA	GCTGCACAGTCGTGACAAAT	[8]
**MDH2**	CGACCTGTTCAACACCAATG	TCCACAACACCTTCCTTTCC	[8]
**OGDH**	AAGTCTAGTGAGAATGGCGTGGACT	CAAGGTAATGTTCCTGTCGGTGAC	[9]
**NMNAT2**	CTCAATACTCCGCAAATACAAAA	CTGGGACAGGTAATCCACAAC	[10]
**NAMPT**	AGCCGAGTTCAACATCCTCCT	AGACATCTTTGGCTTCCTGGAT	[11]
**NRK1**	AAGCCCCTTGACACTATATGGA	TCTGGAGGCTGATAGACCCT	[12]

Total RNA Sequencing

Total RNA sequencing libraries were prepared according to the manufacturer’s instructions (Illumina Truseq Stranded Total RNA Library prep kit with Ribo-zero Human/mouse/rat). 400 ng of total RNA was subjected to ribosomal RNA depletion, with Ribo-zero Human/mouse/rat reagent using biotinylated probes that selectively bind rRNA species. The quality of the amplified libraries was verified by capillary electrophoresis (Bioanalyzer, Agilent). After qPCR using SYBR Green PCR Master Mix (Applied Biosystems, Waltham, MA), we combined libraries that index tagged in equimolar amounts in the pool. RNA sequencing was performed using an Illumina NovaSeq 6000 system according to the manufacturer's instructions for 2 x 100 sequencing.

Reads for each sample were mapped to the reference genome (Human GRCh37(Genome Reference Consortium Human Build 37)-hg19) by Tophat (v2.0.13) (http://ccb.jhu.edu/software/topath). The aligned results were added to Cuffdiff (v2.2.1) (http://cole-trapnell-lab.github.io/cufflinks/papers/) to calculate FPKM (Fragments Per Kilobase of transcript per Million) value and to report differentially expressed genes. For library normalization and dispersion estimation, geometric and pooled methods were applied (http://cole-trapnell-lab.github.io/cufflinks/cuffdiff/). Using one of Cuffdiff outputs, “gene_exp.diff”, (Differentially Expressed Genes (DEGs) were identified. To detect DEGs between group 1 as control and group 2 as case, two filtering processes were applied. First, using Cuffdiff status code, genes that only have “OK” status were extracted. Status code indicates whether each condition contains enough reads in a locus for a reliable calculation of expression level, and “OK” status means the test is successful to calculate gene expression level. For the second filtering, 2-fold change was calculated and genes belonging to the following range were selected (Up-regulated: log2[case]-log2[control] > log2(2) =1: Down-regulated: log2[case]-log2[control] < log2(1/2) =-1). All the data from DEG analysis were confirmed using Ingenuity Pathway Analysis (IPA, Qiagen, Redwood city, CA) and further analyzed for functional networking study, or group comparison. Heat maps for unsupervised hierarchical clustering were generated using MultiExperimental Viewer (MeV), a Java-based application system [13]. Raw count data were converted to FPKM values and differential expression detection and functional annotation enrichment detection were performed. For the comparison between Ctrl and LOAD, genes were considered differentially expressed if FDR<0.05.

Immunocytochemistry

Cells were plated in 24 well dishes on glass coverslips, fixed in 4 % paraformaldehyde (PFA; Thermo Fischer, 28906) in 1 x PBS for 30 min and stored at 4 °C until being used. Immunocytochemistry was performed by permeabilizing the cells with 0.1 % Triton-X (Sigma, T9284-100ML) + 10 % Normal Donkey Serum (NDS; Jackson Labs, cat # 017-000-121). The following primary antibodies and dilutions where used: SOX1 (Millipore, AB15766, Rabbit Polyclonal, 1:500), PAX6 (BioLegend, 901301, Rabbit Polyclonal, 1:500), NESTIN (R&D Systems, MAB1259, Mouse Monoclonal, 1:500), GFAP (Dako, Z0334, Rabbit

Polyclonal, 1:500), GLAST (Alomone Labs, AGC-021, Rabbit Polyclonal, 1:100), and S100ß (Sigma, S2532, Mouse Monoclonal, 1:500).

For quantifying GLUT1, INSR, and IGF-1R, cells were plated at low density, i.e., 1 x 10^3 cells/well for astrocytes and 5 x 10^3 cells/well for NPCs. Immunocytochemistry was performed by permeabilizing the cells with 0.2 % Tween-20 (Sigma, P1379) in 1 x PBS, followed by DAKO Protein Block, serum-free solution (Agilent, X0909). The following primary antibodies and dilutions where used: INSR Alpha (Invitrogen, MA5-13759, Mouse Monoclonal, clone 83-14, 1:100), IGF1-R (Santa Cruz Biotechnologies, sc-462, Mouse Monoclonal, clone 3B7, 1:100) and GLUT-1 (Novus Biologicals, NB110-39113, Rabbit Polyclonal, 1:100).

Following primary antibody, cells were incubated with goat anti-mouse Alexa Fluor 488 (Invitrogen, A-11001, 1:1000) and goat anti-rabbit Alexa Fluor 568 (Invitrogen, A-11011, 1:1000) secondary antibodies. Hoechst 33342 (5 μg/ml) was subsequently used as nuclear counterstain (Thermo Fisher Molecular Probes, H1399). Coverslips were mounted on Superfrost Plus glass slides (VWR, 48311-703) using Vectashield vibrance antifade mounting medium (Vector Labs, H-1700). Slides were allowed to dry overnight before being imaged. Cells were visualized using an Axiovert 200M inverted microscope (Carl Zeiss, Inc.) and captured with the Zeiss Axiocam 503 Mono and Zeiss Zen Lite program.

CellProfiler

CellProfiler is a free program and can be downloaded and installed from the following website (www.cellprofiler.org). For the current study we used version CellProfiler 3.1.8. Cells were imaged at 100 x using oil-immersion and 10 images from each slide were analyzed per subject. We focused on the following modules: (a) *IdentifyPrimaryObjects,* which recognizes biological objects of interest and from which used the following data; *Count:* Total number of primary objects identified in each image. (b) MeasureObjectSizeShape, which measures the area and shape of identified objects and from which we used; *Area:* The number of pixels in a given region. We then normalized the total numbers of speckles (counts) to the cell area using green for INSR or IGF1-R and red for GLUT-1.

Statistical analysis

Data were plotted as mean ± standard error of the mean (SEM) from at least 2 independent experiments performed in triplicates (n = 3), unless otherwise stated. One-way analysis of variance (ANOVA) tests for independent measures were performed using the Social Science Statistics software (http://www.socscistatistics.com/Default.aspx) or PRISM 8 for macOS Version 8.1.0. Differences of comparison were considered statistically significant when p-values were less than 0.05, while p-values between 0.05 and 0.1 were considered trend data.

Supplementary Table 1 List of iPSC lines and donor information.

Sample	Age	Sex	Race	Diagnosis	Donor Cell	Karyotype	APOE Genotype	iPSC Characterization	iPSC Source	Methodology	HIS	MOCA	MMSE	Comorbidity
C1	21	M	Asian	Ctrl	Dermal Fibroblast	normal, male	E2/3	McPhie et al. 2018	NYSCF	RNA Transfection				no serious illness
C2	37	M	white	Ctrl	Dermal Fibroblast	normal, male	E3/3	Yoshimizu et al., 2015	Tsai Lab	RNA Transfection				no serious illness
C3	47	M	white	Ctrl	Dermal Fibroblast	normal, male	E3/4	Score card, Teratoma	UNC	Sendai Virus				no serious illness
C4	60	F	white	Ctrl	Dermal Fibroblast	normal, female	E3/3	Score card, Teratoma	UNC	Sendai Virus				no serious illness
C5	61	M	white	Ctrl	Dermal Fibroblast	normal, male	E3/4	Score card, Teratoma	UNC	Sendai Virus				no serious illness
AD1	56	M	white	AD	Dermal Fibroblast	normal, male	E3/3	Teratoma	Tsai Lab	Sendai Virus	0	24		no serious illness
AD2	65	M	white	AD	Dermal Fibroblast	normal, male	E3/4	Teratoma	Tsai Lab	Sendai Virus	NA	NA		Glaucoma, hypertension, CAD, cerebrovascular accident
AD3	71	F	white	AD	Dermal Fibroblast	normal, female	E4/4	Teratoma	Tsai Lab	Sendai Virus	2	10		no serious illness
AD4	76	M	white	AD	Dermal Fibroblast	normal, male	E3/4	Teratoma	Tsai Lab	Sendai Virus	1	13		hemochromatosis
AD5	79	M	white	AD	Dermal Fibroblast	normal, male	E4/4	Teratoma	Tsai Lab	Sendai Virus	4	18		Diabetes II, hyperlipidemia, hypertension, CAD
AD6	81	M	white	AD	Dermal Fibroblast	normal, male	E3/4	Teratoma	Tsai Lab	Sendai Virus	1	5		No serious illness
AD7	71	M	white	AD	PBMC	normal, male	E3/4	Score card, Teratoma	UNC	Sendai Virus	0		23	no serious illness
AD8	78	F	white	AD	PBMC	normal, female	E3/4	Score card, Teratoma	UNC	Sendai Virus	2		27	Diabetes II, Hypertension, hyperlipidemia
AD9	91	F	white	AD	PBMC	normal, female	E3/4	Score card, Teratoma	UNC	Sendai Virus	MRI	3		no serious illness

HIS: Hachinsky Score
MOCA: Montreal Cognitive assessment Test
MMSE: Mini-Mental State Exam
CAD: Coronary Artery Disease
MRI: Magnet Resonance Imaging
PBMC: Peripheral Blood Mononuclear Cells

Supplementary Table 2 Raw data of RNA-Seq results.

Microsoft xls file of RNA-Seq data.

Supplementary Table 3 List of RNA-Seq results presented in **Fig. 4d**. Significant changes are in bold and empty boxes indicate no measurement.

	Glycolysis	NPCs		Astrocytes	
**Gene ID**	**Gene Name**	**log2 fold**	**p-value**	**log2 fold**	**p-value**
G6PC3	glucose-6-phosphatase 3	-0.09	0.7897	0.03	0.9299
GALM	N-acetylgalactosamine-6-sulfatase isoform 1 precursor	-0.31	0.4780	0.06	0.8908
**HK1**	hexokinase-1	-0.28	0.3910	**-0.55**	**0.0693**
**HK2**	hexokinase-2	**0.56**	**0.0953**	0.16	0.6574
**PFKFB3**	6-phosphofructo-2-kinase/fructose-2, 6-bisphosphatase 3	-0.36	0.2385	**-0.97**	**0.0026**
PGD	6-phosphogluconate dehydrogenase, decarboxylating	0.19	0.5107	0.37	0.2497
**PGM1**	phosphoglucomutase-1	**-0.59**	**0.0947**	-0.43	0.1454
PGM2	phosphoglucomutase-2	-0.11	0.7415	-0.10	0.7609
**PGM2L1**	glucose 1,6-bisphosphate synthase	**0.78**	**0.0074**	**-0.86**	**0.0118**
PGM3	phosphoacetylglucosamine mutase	-0.48	0.2130	-0.26	0.5077
PGM5	phosphoglucomutase-like protein 5	-0.45	0.4764	-3.16	1.0000
PGM5-AS1	phosphoglucomutase-like protein 5, antisense	-1.92	0.2232	1.39	1.0000
PGM5P2	phosphoglucomutase 5 pseudogene 2	0.12	0.8700	0.17	0.7225
PYGB	glycogen phosphorylase, brain form	-0.02	0.9364	0.41	0.3174
**PYGL**	glycogen phosphorylase, liver form	**-1.27**	**0.0002**	-0.17	0.5697
PYGM	glycogen phosphorylase, muscle form	0.09	1.0000	0.20	1.0000

	CAC	NPCs		Astrocytes	
**Gene ID**	**Gene Name**	**log2 fold**	**p-value**	**log2 fold**	**p-value**
ACO1	1-aminocyclopropane-1-carboxylate oxidase 1	-0.11	0.7146	0.15	0.5801
ACO2	1-aminocyclopropane-1-carboxylate oxidase 2	-0.42	0.2830	0.04	0.9291
**FAHD2A**	fumarylacetoacetate hydrolase domain-containing protein 2	-0.16	0.6633	**-0.82**	**0.0143**
FH	fumarate hydratase, mitochondrial	0.17	0.5539	-0.17	0.5750
GPT	alanine aminotransferase 1	-0.05	1.0000	0.15	1.0000
GPT2	alanine aminotransferase 2	0.30	0.3052	-0.31	0.3333
**IDH1**	Isocitrate dehydrogenase [NAD] regulatory subunit 1	**0.56**	**0.0763**	0.09	0.7506
IDH1-AS1	Isocitrate dehydrogenase [NAD] regulatory subunit 1, antisense	-0.04	0.9692	-0.60	0.5166
IDH2	Isocitrate dehydrogenase [NAD] regulatory subunit 2	0.37	0.1924	0.15	0.6109
IDH3A	isocitrate dehydrogenase [NAD] subunit alpha	-0.05	0.8989	-0.10	0.7669
IDH3B	isocitrate dehydrogenase [NAD] subunit beta	0.05	0.8787	-0.07	0.8027
IDH3G	isocitrate dehydrogenase [NAD] subunit gamma	-0.36	0.5226	0.03	0.9514
**LDHA**	L-lactate dehydrogenase A chain	**-0.93**	**0.0025**	**-1.02**	**0.0042**
LDHAL6A	L-lactate dehydrogenase A-like 6A	-0.17	1.0000	-0.19	1.0000
LDHAL6B	L-lactate dehydrogenase A-like 6B	-0.45	1.0000	-1.01	0.4378
LDHB	L-lactate dehydrogenase B chain	0.14	0.6681	0.16	0.6305
LDHC	L-lactate dehydrogenase C chain				
LDHD	probable D-lactate dehydrogenase, mitochondrialial	-0.79	0.3820	-0.16	1.0000
MDH1	malate dehydrogenase subunit 1	-0.10	0.7564	-0.14	0.6310
MDH1B	putative malate dehydrogenase 1B	-0.04	0.9770	-0.02	1.0000
MDH2	malate dehydrogenase subunit 2	0.11	0.6869	0.00	0.9984
**ME1**	NADP-dependent malic enzyme	**-1.73**	**0.0003**	**-0.73**	**0.0527**
**ME3**	NADP-dependent malic enzyme, mitochondrial	**-1.67**	**0.0050**	0.13	0.7912
OGDH	2-oxoglutarate dehydrogenase, mitochondrial	-0.16	0.5731	0.06	0.8359
OGDHL	2-oxoglutarate dehydrogenase-like, mitochondrial	0.03	0.9504	0.79	1.0000
PDHA1	pyruvate dehydrogenase E1 component subunit alpha, somatic form	0.10	0.7536	-0.04	0.8795
PDHB	pyruvate dehydrogenase E1 component subunit beta	-0.36	0.2324	0.14	0.6157
**PDK3**	mitochondrial pyruvate dehydrogenase E1alpha-kinase 3	0.08	0.7999	**-1.95**	**0.0001**
**PDPR**	p53 and DNA damage-regulated protein 1	0.15	0.6231	**0.68**	**0.0257**
SDHA	succinate dehydrogenase [ubiquinone] flavoprotein subunit, mitochondrial	0.13	0.6641	-0.10	0.7400
SDHAF1	succinate dehydrogenase assembly factor 1, mitochondrial	-0.02	0.9638	0.01	0.9897
SDHAF2	succinate dehydrogenase assembly factor 2, mitochondrial	-0.17	0.6187	0.00	0.9921
SDHAF3	succinate dehydrogenase assembly factor 3, mitochondrial	0.18	0.6885	0.00	0.9970
SDHAF4	succinate dehydrogenase assembly factor 4, mitochondrial	-0.08	0.8989	0.01	0.9828
SDHAP1	succinate dehydrogenase complex flavoprotein subunit A pseudogene 1	0.31	0.5168	0.27	0.6207
SDHAP2	succinate dehydrogenase complex flavoprotein subunit A pseudogene 1	0.27	0.5593	0.57	0.3116
SDHAP3	succinate dehydrogenase complex flavoprotein subunit A pseudogene 1	-0.05	0.9254	-0.02	1.0000
SDHB	succinate dehydrogenase [ubiquinone] iron-sulfur subunit, mitochondrial	-0.14	0.6594	-0.08	0.7743
SDHC	succinate dehydrogenase cytochrome b560 subunit, mitochondrial	-0.11	0.7383	-0.15	0.6173
SDHD	succinate dehydrogenase [ubiquinone] cytochrome b small subunit, mitochondrial	-0.21	0.52	0.03	0.9111

	ETC	NPCs		Astrocytes	
**Gene ID**	**Gene Name**	**log2 fold**	**p-value**	**log2 fold**	**p-value**
**CYBRD1**	cytochrome b reductase 1	**-0.65**	**0.0727**	**0.62**	**0.0450**
**CYP1B1**	cytochrome P450 1B1	**-1.34**	**0.0021**	**0.64**	**0.1048**
**CYP26B1**	cytochrome P450 26B1, retinoic acid hydroxylase	**3.14**	**0.0007**	-1.98	1.0000
**CYP27A1**	cytochrome P450 27A1, sterol 26-hydoxylase	**-1.93**	**0.0010**	0.56	0.2212
**CYP27C1**	cytochrome P450 27C1	0.62	0.2249	**-0.98**	**0.0916**
**UQCRHL**	cytochrome b-c1 complex subunit 6-like, mitochondrial	**1.67**	**0.0851**	-0.63	1.0000

	G3P shuttle	NPCs		Astrocytes	
**Gene ID**	**Gene Name**	**log2 fold**	**p-value**	**log2 fold**	**p-value**
**GPD1**	glycerol-3-phosphate dehydrogenase (NAD(+))	**-7.34**	**0.0126**	0.17	1.0000
**GPD1L**	glycerol-3-phosphate dehydrogenase 1-like protein	**-0.61**	**0.0999**	-0.34	0.3186
**GPD2**	glycerol-3-phosphate dehydrogenase (NAD(+)), mitochondrial	**-1.59**	**0.0001**	-0.15	0.6302

	L-ornithine	NPCs		Astrocytes	
**Gene ID**	**Gene Name**	**log2 fold**	**p-value**	**log2 fold**	**p-value**
**CPS1**	Gly-Xaa carboxypeptidase//carbamoyl-phosphate synthase, mitochondrial	-0.61	0.1140	**0.63**	**0.0756**
CPS1-IT1	Gly-Xaa carboxypeptidase//carbamoyl-phosphate synthase, intronic transcript	0.35	1.0000	0.89	1.0000
**ODC1**	mitochondrial 2-oxodicarboxylate carrier/ornithine decarboxylase	**0.61**	**0.0512**	0.07	0.8318

	beta-Oxidation, MAS	NPCs		Astrocytes	
**Gene ID**	**Gene Name**	**log2 fold**	**p-value**	**log2 fold**	**p-value**
ABAT	4-aminobutyrate aminotransferase	0.50	0.1397	-0.11	0.7922
**ABHD3**	abhydrolase domain containing 3, hospholipase	**0.83**	**0.0436**	-0.09	0.8806
**ABHD4**	abhydrolase domain containing 4, hospholipase	**-1.14**	**0.0018**	-0.10	0.7390
**ACOT1**	acyl-coenzyme A thioesterase 1	**-4.28**	**0.0154**	-1.03	0.4413
**ACOT2**	acyl-coenzyme A thioesterase 2	**-2.85**	**0.0012**	-0.46	0.3091
**ACOT7**	acyl-coenzyme A thioesterase 7	**0.55**	**0.1000**	-0.36	0.2891
**ACOT9**	acyl-coenzyme A thioesterase 9	**-0.99**	**0.0107**	-0.42	0.1844
**ACOX2**	peroxisomal acyl-coenzyme A oxidase 2	**-1.78**	**0.0418**	1.12	0.1352
ACSBG1	long-chain-fatty-acid--CoA ligase, member 1	**-0.93**	**0.0658**	0.24	1.0000
ACSBG2	long-chain-fatty-acid--CoA ligase, member 2	0.40	1.0000	0.28	1.0000
**ACSF2**	acyl-CoA synthetase family, member 2	-0.16	0.6751	**0.70**	**0.0530**
ACSF3	acyl-CoA synthetase family, member 3	-0.03	0.9317	0.16	0.6721
ACSL1	long-chain-fatty-acid--CoA ligase 1	-0.12	0.7529	-0.43	0.2014
ACSL3	long-chain-fatty-acid--CoA ligase 3	-0.43	0.1811	0.00	0.9938
**ACSL4**	long-chain-fatty-acid--CoA ligase 4	**-0.65**	**0.0536**	-0.40	0.2538
ACSL5	long-chain-fatty-acid--CoA ligase 5	-0.07	1.0000	-0.07	1.0000
ACSL6	long-chain-fatty-acid--CoA ligase 6	-0.64	0.2743	1.07	1.0000
ACSM1	acyl-coenzyme A synthetase, member 1				
ACSM2A	acyl-coenzyme A synthetase, member 2A				
ACSM2B	acyl-coenzyme A synthetase, member 2B				
ACSM3	acyl-coenzyme A synthetase, member 3	1.00	1.0000	1.14	1.0000
ACSM4	acyl-coenzyme A synthetase, member 4				
ACSM5	acyl-coenzyme A synthetase, member 5				
ACSM6	acyl-coenzyme A synthetase, member 6	-3.35	1.0000		
ACSS1	acetyl-coenzyme A synthase short chain family member 1	-0.65	0.1208	1.25	0.3070
ACSS2	acetyl-coenzyme A synthase short chain family member 2	0.11	0.7299	0.24	0.4166
ACSS3	acetyl-coenzyme A synthase short chain family member 3	**-3.33**	**0.0001**	-0.77	0.1336
**BBOX1**	gamma-butyrobetaine dioxygenase	**-2.12**	**0.0133**	-0.68	1.0000
**BCAT1**	branched-chain amino acid aminotransferase 1	**0.66**	**0.0908**	0.05	0.8822
BCAT2	branched-chain amino acid aminotransferase 2	0.17	0.6889	0.14	0.6893
BCKDHA	branched Chain Keto Acid Dehydrogenase E1 Subunit Alpha	-0.01	0.9854	-0.12	0.7442
BCKDHB	branched Chain Keto Acid Dehydrogenase E1 Subunit Beta	0.12	0.7546	0.06	0.8846
BDH1	3-hydroxybutyrate dehydrogenase type 1	-0.22	0.7214	-0.36	0.5480
BDH2	3-hydroxybutyrate dehydrogenase type 2	-0.08	0.8130	-0.03	0.9250
**CPT1A**	carnitine O-palmitoyltransferase 1, liver isoform	0.57	0.1952	**1.29**	**0.0019**
CPT1B	carnitine O-palmitoyltransferase 1, muscle isoform	-0.18	0.7079	-0.62	0.5085
CPT1C	carnitine O-palmitoyltransferase 1, brain isoform	-0.23	0.5314	-0.41	0.3388
CPT2	carnitine O-palmitoyltransferase 2	-0.23	0.5845	0.00	0.9954
CPTP	ceramide-1-phosphate transfer protein	-0.23	0.5710	-0.33	0.3597
CROT	peroxisomal carnitine O-octanoyltransferase	-0.48	0.3044	-0.42	0.3188
**ECI2**	enoyl-CoA delta isomerase 2	**-0.66**	**0.0468**	-0.22	0.4748
**EHHADH**	peroxisomal bifunctional enzyme	-0.38	0.5356	**-1.89**	**0.0185**
GLUD1	glutamate dehydrogenase 1, mitochondrial	0.05	0.8525	0.06	0.8302
GLUD1P3	glutamate dehydrogenase 1, mitochondrial, pseudogene 3	0.05	0.9140	0.38	0.4890
GLUD1P7	glutamate dehydrogenase 1, mitochondrial, pseudogene 7	0.03	0.9687	0.51	1.0000
GLUD2	glutamate dehydrogenase 2, mitochondrial	0.09	1.0000	-0.93	1.0000
GLUL	glutamine synthetase/glutamine synthetase isoform 1	0.14	0.6685	-0.43	0.2008
HPD	4-Hydroxyphenylpyruvate Dioxygenase	-1.20	1.0000	-4.60	0.2424
HPDL	4-Hydroxyphenylpyruvate Dioxygenase-like	-0.24	0.7953	-0.34	1.0000
**HSD11B2**	corticosteroid 11-beta-dehydrogenase isozyme 2	**1.38**	**0.0524**	-0.07	1.0000
SLC22A5	solute Carrier Family 22 Member 5	-0.51	0.5156	-0.03	0.9634
SLC25A12	solute Carrier Family 25 Member 12	-0.39	0.2470	0.25	0.4159

	Others	NPCs		Astrocytes	
**Gene ID**	**Gene Name**	**log2 fold**	**p-value**	**log2 fold**	**p-value**
IGF1	insulin-like growth factor I	-2.63	1.0000	0.51	1.0000
IGF1R	insulin-like growth factor 1 receptor	0.17	0.6088	0.16	0.6193
**IGF2**	insulin-like growth factor II	**-2.49**	**0.0550**	**0.70**	**0.0906**
IGF2-AS	insulin-like growth factor II, antisense			1.71	1.0000
INS	insulin precursor				
INS-IGF2	insulin, isoform 2 precursor				
INSR	insulin receptor isoform long preproprotein	0.10	0.7490	0.28	0.4324
INSRR	insulin receptor-related protein precursor	-5.34	1.0000	-0.20	1.0000
IRS1	insulin receptor substrate 1	-0.45	0.2232	-0.44	0.1212
**IRS2**	insulin receptor substrate 2	**-1.07**	**0.0006**	**-0.84**	**0.0041**
IRS4	insulin receptor substrate 4	-3.41	0.2609	-0.92	1.0000
**SLC2A1**	solute carrier family 2, facilitated glucose transporter member 1	**1.04**	**0.0025**	0.23	0.4982
SLC2A1-AS1	solute carrier family 2, facilitated glucose transporter member 1, antisense	0.04	0.9768	-0.56	0.6433
**SLC2A10**	solute carrier family 2, facilitated glucose transporter member 10	**-1.02**	**0.0054**	-0.06	0.8542
SLC2A11	solute carrier family 2, facilitated glucose transporter member 11	0.16	0.8167	-0.09	0.8945
SLC2A12	solute carrier family 2, facilitated glucose transporter member 12	0.55	0.1309	**1.19**	**0.0102**
**SLC2A13**	proton myo-inositol cotransporter	**-0.63**	**0.0937**	-0.42	0.3126
SLC2A14	solute carrier family 2, facilitated glucose transporter member 14	1.15	0.2962	0.96	0.3560
SLC2A2	solute carrier family 2, facilitated glucose transporter member 2	-0.79	1.0000	0.37	1.0000
**SLC2A3**	solute carrier family 2, facilitated glucose transporter member 3	**0.58**	**0.0537**	-0.07	0.7946
SLC2A4	solute carrier family 2, facilitated glucose transporter member 4	0.22	1.0000	0.54	1.0000
SLC2A4RG	solute carrier family 2, facilitated glucose transporter member 4 regulator	-0.36	0.4945	0.13	0.7093
SLC2A5	solute carrier family 2, facilitated glucose transporter member 5	-3.09	1.0000	-0.54	1.0000
SLC2A6	solute carrier family 2, facilitated glucose transporter member 6	-0.28	0.4450	-0.32	0.3620
SLC2A7	solute carrier family 2, facilitated glucose transporter member 7				
SLC2A8	solute carrier family 2, facilitated glucose transporter member 8	0.00	0.9990	-0.59	0.2893
SLC2A9	solute carrier family 2, facilitated glucose transporter member 9	-0.25	1.0000	-0.73	1.0000

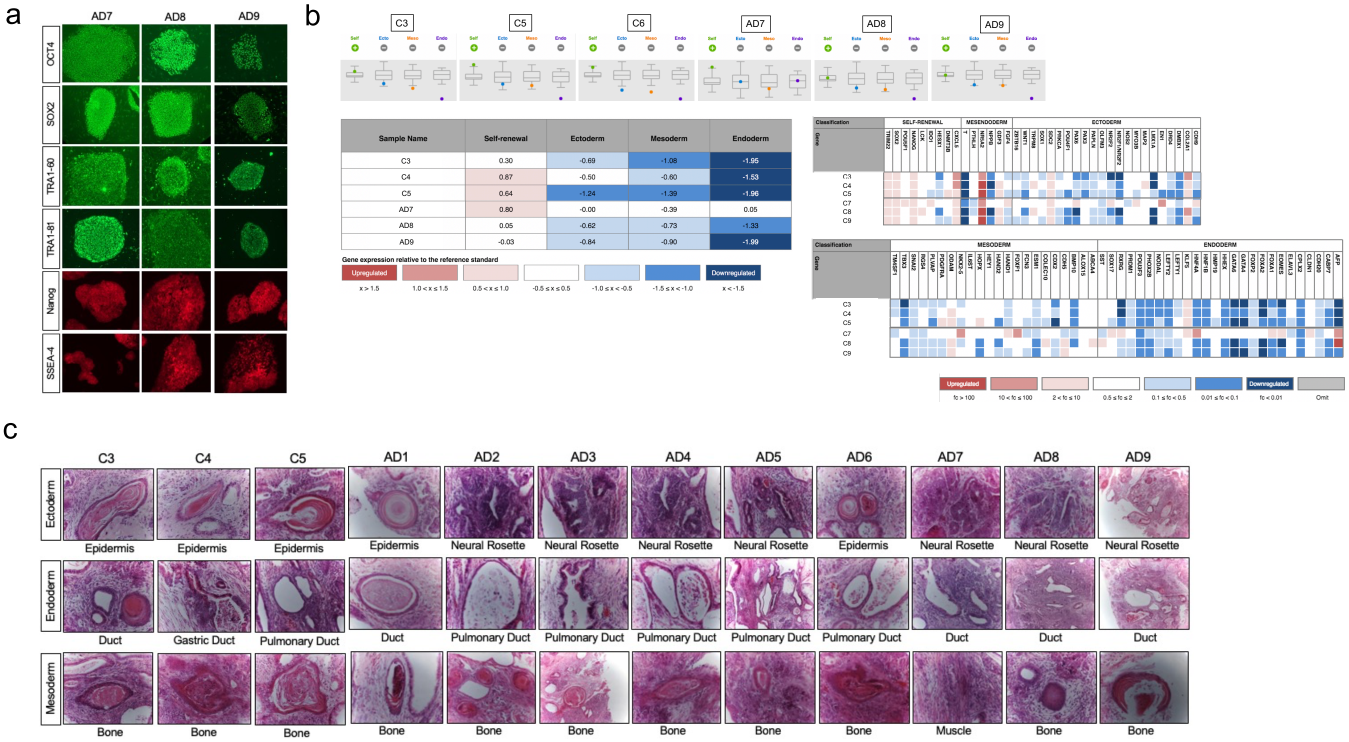

Supplementary Fig. 1 Human iPSC lines are pluripotent. a Immunostaining of iPSC lines AD7, AD8, and AD9 with common pluripotency markers OCT4, SOX2, TRA-60, TRA1-81, NANOG, and SSEA-4. **b** TaqMan hPSC scorecard assays for lines C3, C4, C5, AD7, AD8, and AD9. **c** Upon injection into SCID mice, Control- and LOAD-iPS cells form teratomas *in vivo*, which contain tissues of all three embryonic germ layers, such as epidermis and neural epithelium (ectoderm), bone tissue (mesoderm), and epithelium duct (endoderm).

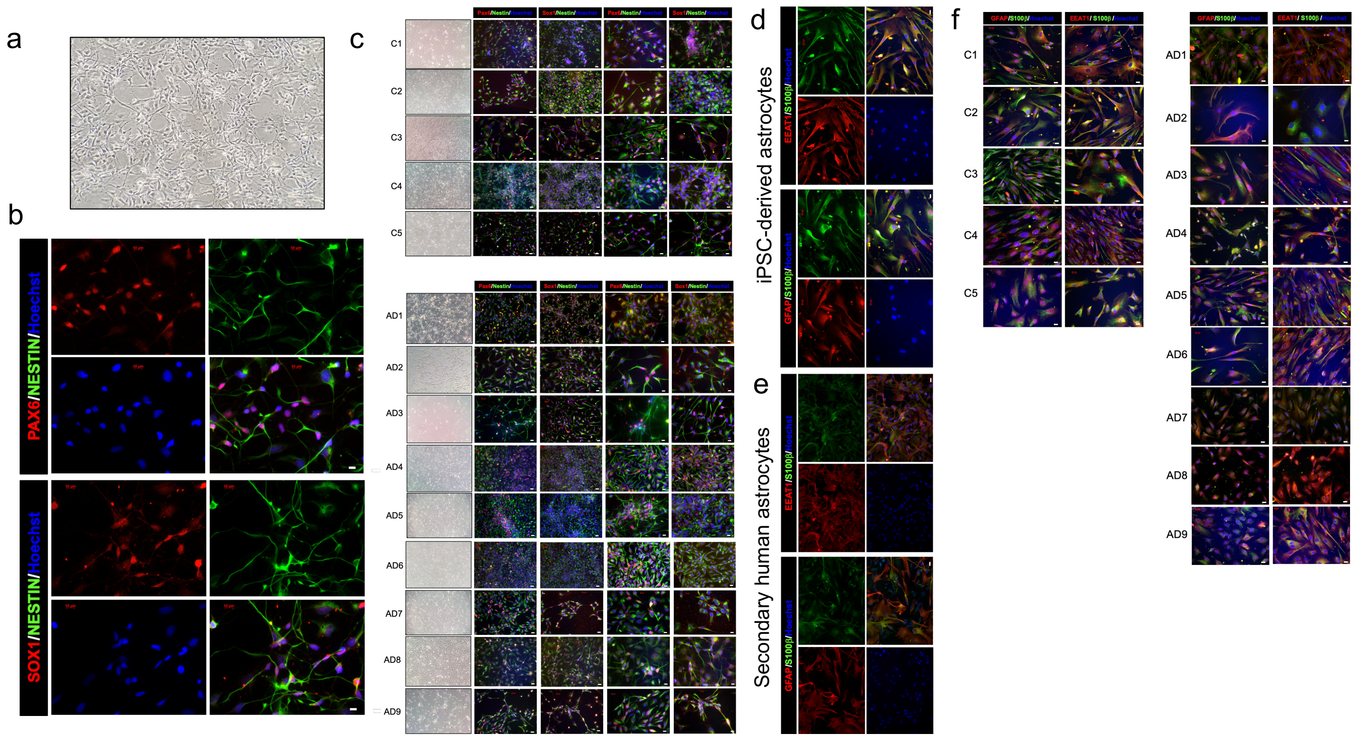

Supplementary Fig. 2 Differentiation of iPSCs to NPCs and astrocytes. a-f Morphological characterization of iPSC-differentiated NPCs (**a**-**c**) and astrocytes (**d**-**f**). Brightfield images (**a**, **c**) and immunocytochemistry of NPCs expressing PAX6, SOX1, and NESTIN (**b**, **c**), and astrocytes expressing GFAP, S100β, and EEAT1 (**d**, **e**, **f**). Hoechst was used to stain nuclei. Scale bars, 20 μm in (**b**) to (**f**), and 200 μm in left panels of (**c**) for NPCs AD4, AD5, and AD6.

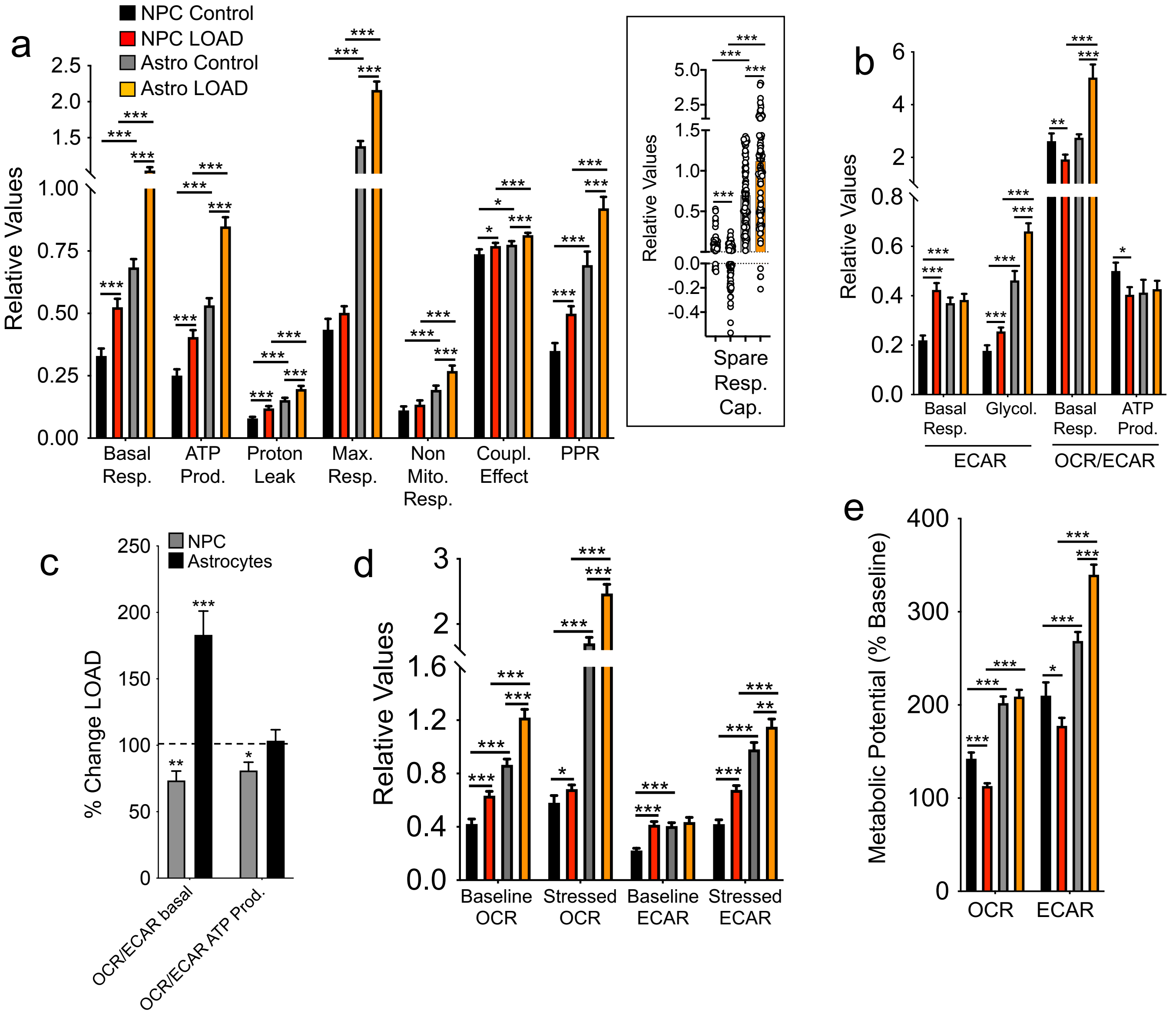

Supplementary Fig. 3 LOAD and Control NPCs and astrocytes exhibit differences in their bioenergetic profiles. a-c Calculated values of bioenergetic parameters for LOAD and Control NPCs (black and red bars, respectively) and astrocytes (grey and orange bars, respectively) plotted as relative values OCR (pmol/min) (**a**) and ECAR (mpH/min) or OCR/ECAR (**b**), and percent change of LOAD over Control for OCR/EACR (**c**). **d,e** Results from Cell Energy Phenotype Test Report Generator plotted as relative values OCR (pmol/min) or ECAR (mpH/min) (**d**) and their calculated metabolic potentials ((stressed OCR or ECAR / baseline OCR or ECAR) x 100%)) (**e**). Data are means +/- SEM from two repeat experiments. *p < 0.1; **p < 0.05; ***p < 0.01 using one-way ANOVA.

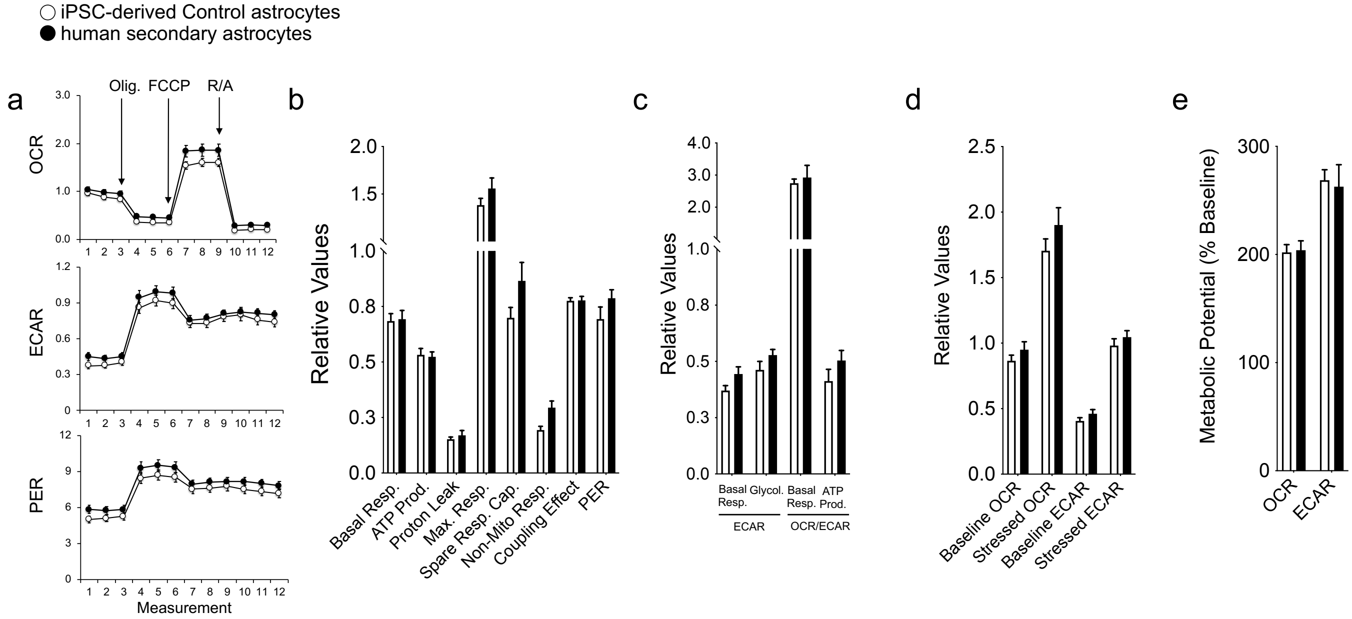

Supplementary Fig. 4 Seahorse data of iPSC-derived Control and secondary human astrocytes. a-e Seahorse data of iPSC-derived Control astrocytes (*n* = 5) and secondary human astrocytes. **a** Profiles of Seahorse XFp Mito Stress Test data for oxidative consumption rates (OCR, pmol/min), extracellular acidification rate (ECAR, mpH/min), and proton efflux rate (PER, pmol H+/min) in secondary human astrocytes. Arrows indicate injections of specific stressors of mitochondrial respiration, including oligomycin (Olig.), carbonyl cyanite-4 (trifluoromethoxy) phenylhydrazone (FCCP), and Rotenone/Antimycin A (R/A). **b**,**c** Calculated values of bioenergetics parameters for OCR (**b**), ECAR, and OCR/ECAR (**c**). **d**,**e** Results from Cell Energy Phenotype Test Report Generator plotted as relative values OCR (pmol/min) or ECAR (mpH/min) (**d**) and their calculated metabolic potentials ((stressed OCR or ECAR / baseline OCR or ECAR) x 100%)) (**e**).

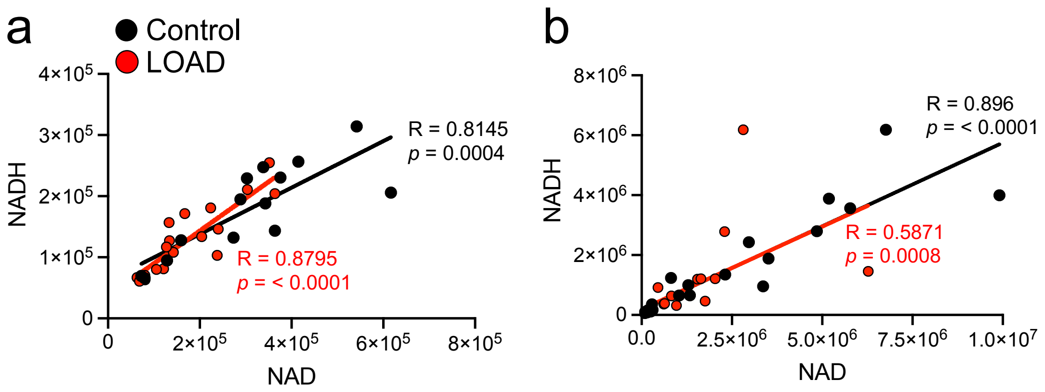

Supplementary Fig. 5 Pearson's correlation coefficients for NAD^+ and NADH. a,**b** Linear correlations for Control (black) and LOAD (red) NPCs (**a**) and astrocytes (**b**) for NAD^+ and NADH, plotted as relative values luminescence. R and *p* values are indicated.

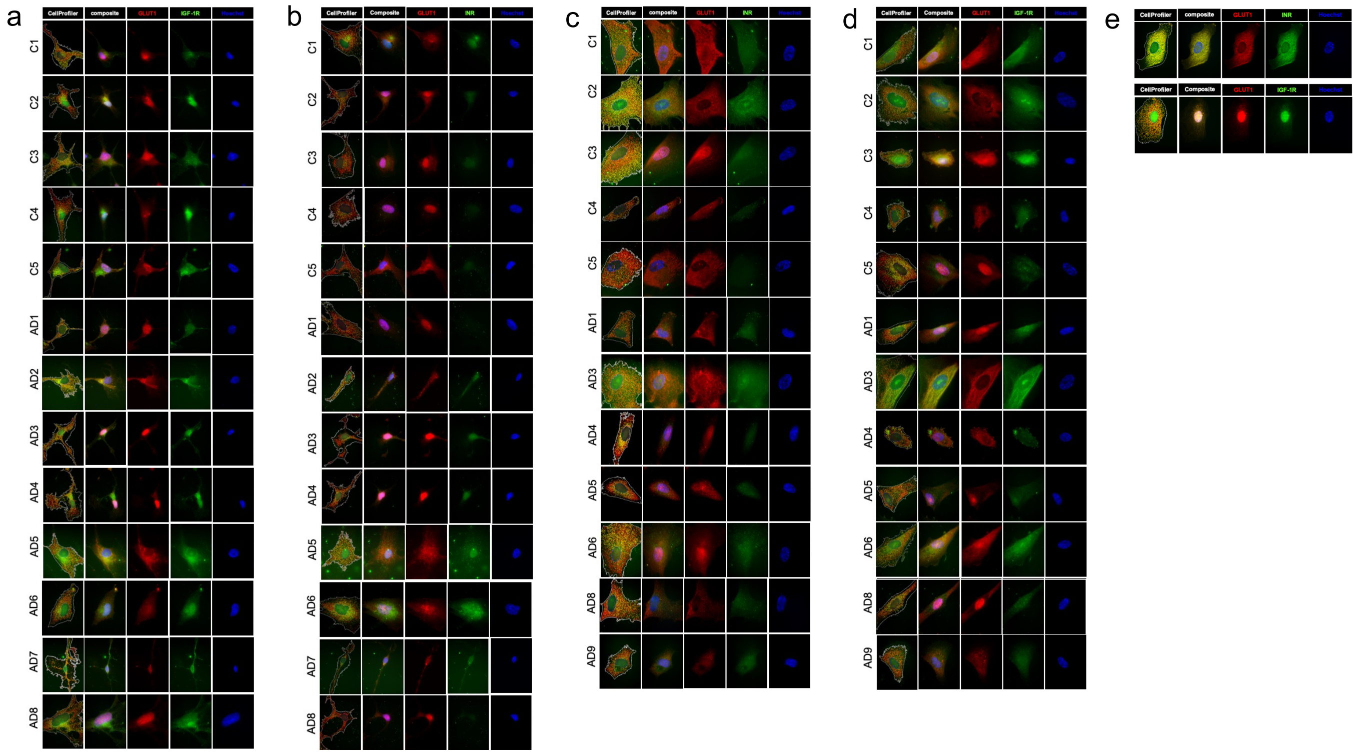

Supplementary Fig. 6 Immunocytochemistry for INR, IGF-1R, and GLUT1. a-e NPCs (**a**,**b**) and astrocytes (**c**,**d**) and secondary human astrocytes (**e**) were stained for INR, IGF-1R, and GLUT1, and CellProfiler images were generated for quantitative analysis of speckles.

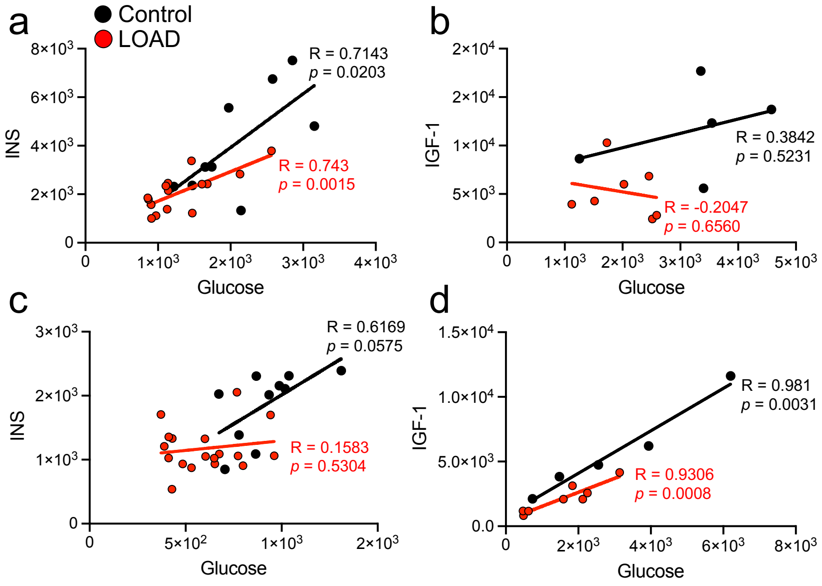

Supplementary Fig. 7 Pearson's correlation coefficients for glucose and INS or IGF-1. a-d Linear correlations for Control (black) and LOAD (red) NPCs (**a**,**b**) and astrocytes (**c**,**d**) for glucose and INS (**a**,**c**), and glucose and IGF-1 (**b**,**d**), plotted as relative values luminescence. R and *p* values are indicated.

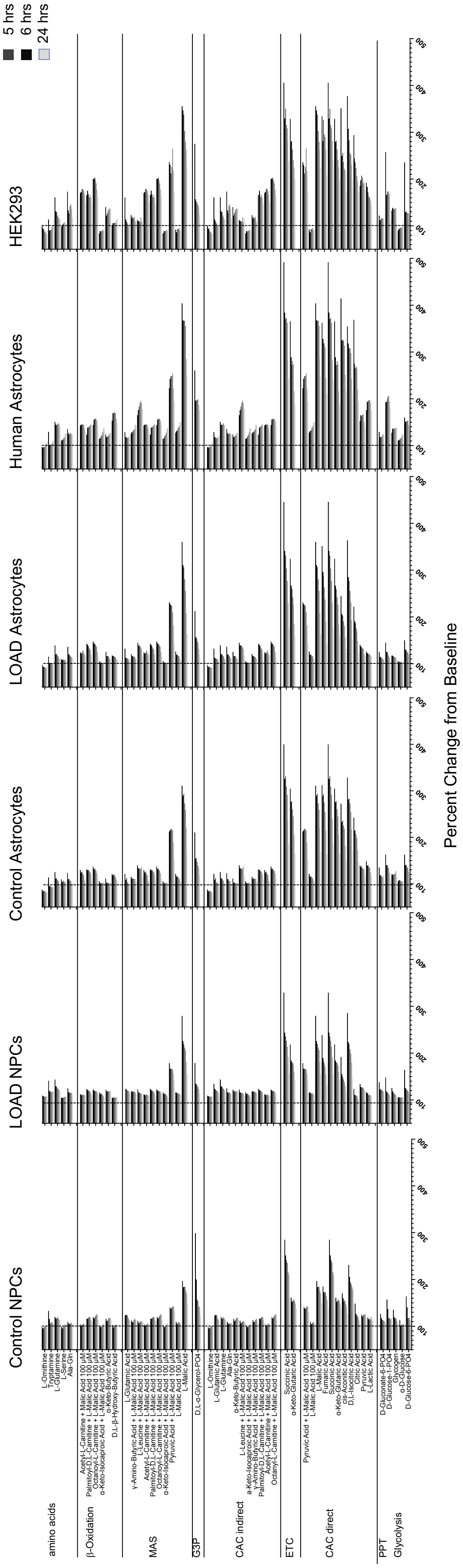

Control NPCs
LOAD NPCs
Control Astrocytes
LOAD Astrocytes
Human Astrocytes
HEK293
1 hrs
2 hrs
3 hrs
4 hrs
5 hrs
6 hrs
24 hrs
amino acids
β-Oxidation
MAS
G3P
CAC indirect
ETC
CAC direct
PPT
Glycolysis
L-Ornithine
Tryptamine
L-Glutamine
L-Serine
Ala-Gln
Succinic Acid
α-Keto-Glutaric Acid
L-Malic Acid
Fumaric Acid
Pyruvic Acid
L-Lactic Acid
Citric Acid
Glycogen
α-D-Glucose
100
200
300
400
500
Percent Change from Baseline

Supplementary Fig. 8 Profiling of cell bioenergetic metabolism in Biolog assays. Data from Biolog experiments on LOAD (*n* = 9) and Control (*n* = 5) NPCs or LOAD (*n* = 9) and Control (*n* = 5) astrocytes, human astrocytes, and HEK293 cells, depicting kinetic measurements at 1 to 6 and 24 hrs. Shown are percent changes of no-substrate normalized values of 31 metabolites.

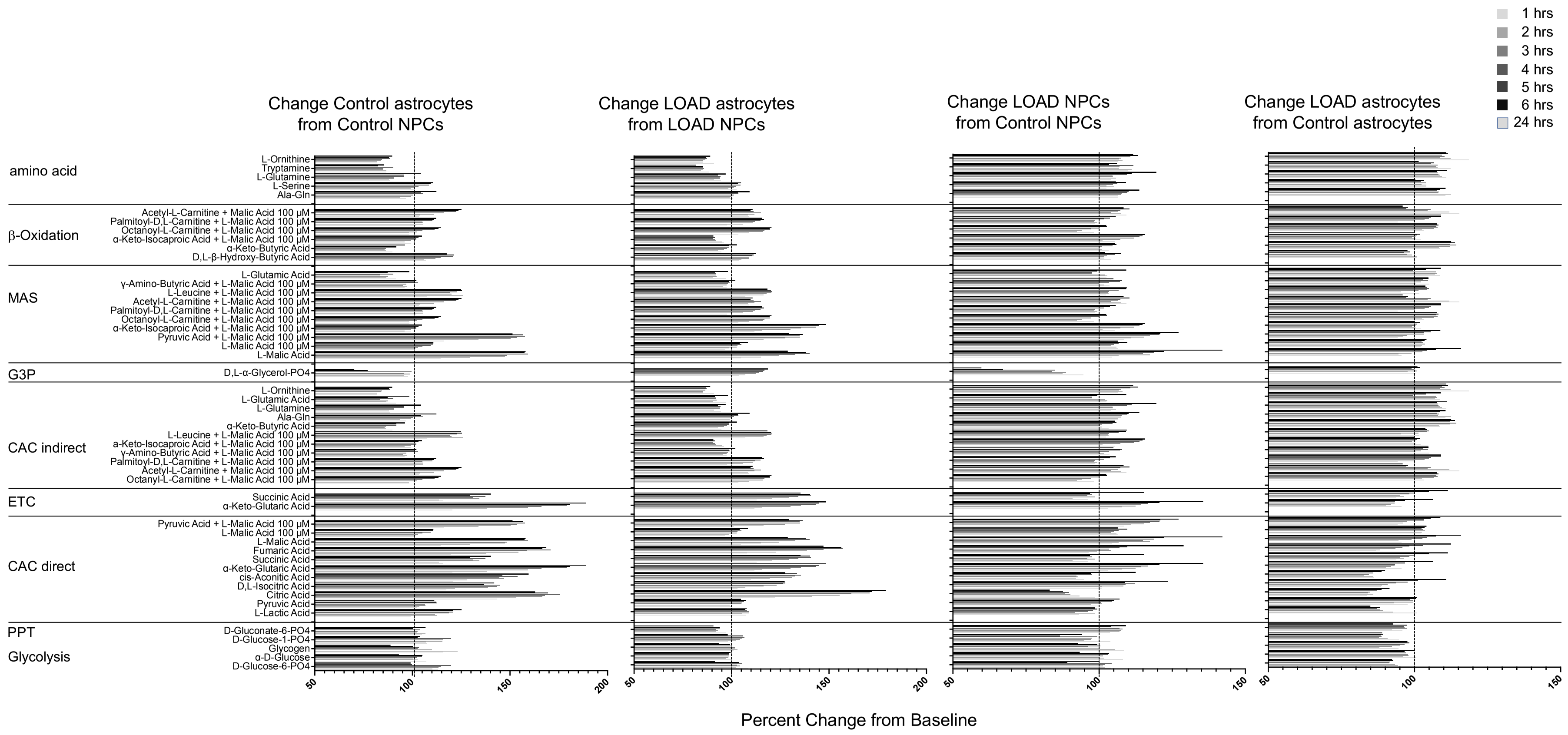

Change Control astrocytes from Control NPCs
Change LOAD astrocytes from LOAD NPCs
Change LOAD NPCs from Control NPCs
Change LOAD astrocytes from Control astrocytes
1 hrs
2 hrs
3 hrs
4 hrs
5 hrs
6 hrs
24 hrs
amino acid
L-Ornithine
Tryptamine
L-Glutamine
L-Serine
Ala-Gln
β-Oxidation
Acetyl-L-Carnitine + Malic Acid 100 μM
Palmitoyl-D,L-Carnitine + L-Malic Acid 100 μM
Octanoyl-L-Carnitine + L-Malic Acid 100 μM
α-Keto-Isocaproic Acid + L-Malic Acid 100 μM
α-Keto-Butyric Acid
D,L-β-Hydroxy-Butyric Acid
MAS
L-Glutamic Acid
γ-Amino-Butyric Acid + L-Malic Acid 100 μM
L-Leucine + L-Malic Acid 100 μM
Acetyl-L-Carnitine + L-Malic Acid 100 μM
Palmitoyl-D,L-Carnitine + L-Malic Acid 100 μM
Octanoyl-L-Carnitine + L-Malic Acid 100 μM
α-Keto-Isocaproic Acid + L-Malic Acid 100 μM
Pyruvic Acid + L-Malic Acid 100 μM
L-Malic Acid 100 μM
L-Malic Acid
G3P
D,L-α-Glycerol-PO4
CAC indirect
L-Ornithine
L-Glutamic Acid
L-Glutamine
Ala-Gln
α-Keto-Butyric Acid
L-Leucine + L-Malic Acid 100 μM
a-Keto-Isocaproic Acid + L-Malic Acid 100 μM
γ-Amino-Butyric Acid + L-Malic Acid 100 μM
Palmitoyl-D,L-Carnitine + L-Malic Acid 100 μM
Acetyl-L-Carnitine + Malic Acid 100 μM
Octanyl-L-Carnitine + L-Malic Acid 100 μM
ETC
Succinic Acid
α-Keto-Glutaric Acid
CAC direct
Pyruvic Acid + L-Malic Acid 100 μM
L-Malic Acid 100 μM
L-Malic Acid
Fumaric Acid
Succinic Acid
α-Keto-Glutaric Acid
cis-Aconitic Acid
D,L-Isocitric Acid
Citric Acid
Pyruvic Acid
L-Lactic Acid
PPT
Glycolysis
D-Gluconate-6-PO4
D-Glucose-1-PO4
Glycogen
α-D-Glucose
D-Glucose-6-PO4
50
100
150
200
50
100
150
200
50
100
150
50
100
150
Percent Change from Baseline

Supplementary Fig. 9 Differences in bioenergetic functions between Control and LOAD cells. Data from Biolog experiments on LOAD (*n* = 9) and Control (*n* = 5) NPCs or LOAD (*n* = 9) and Control (*n* = 5) astrocytes for kinetic measurements at 1 to 6 and 24 hrs. Shown are percent changes of no-substrate control normalized values as percent astrocytes versus NPCs and LOAD versus Control cells of 31 metabolites organized by association with metabolic function.

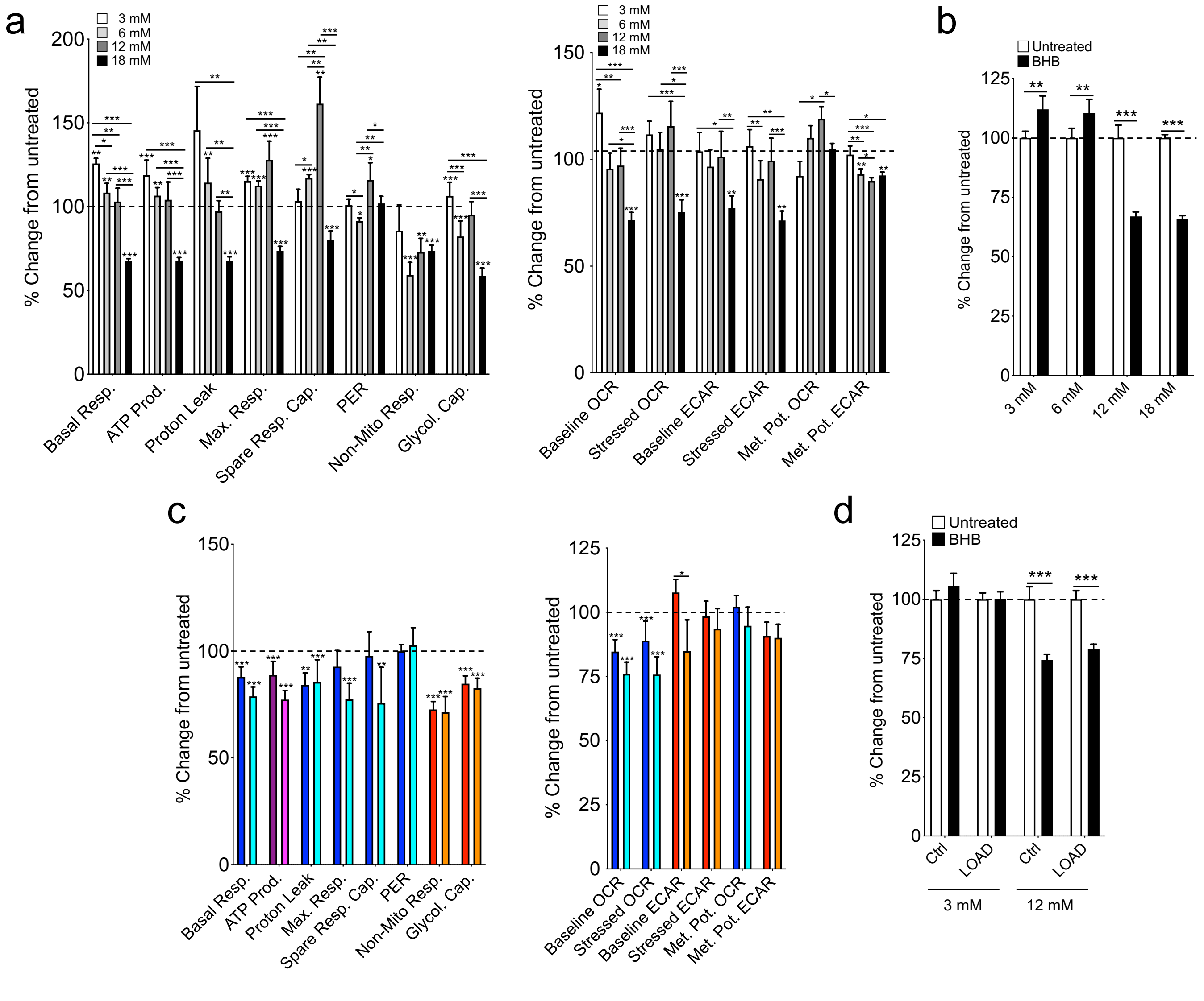

Supplementary Fig. 10 Effects of BHB treatment on bioenergetic functions in astrocytes. a Seahorse data on secondary human astrocytes treated with different doses of BHB and plotted as percent change BHB compared to untreated controls. **b** CyQuant fluorescence measurements on astrocytes after conducting the Seahorse experiments indicating cell viability. Data are plotted as percent change fluorescence comparing BHB-treated from untreated cells. **c** Treatment of Control (*n* = 5) and LOAD (*n* = 6) astrocytes with 12 mM BHB. Seahorse data are plotted as substrate effect, i.e., percent change in BHB-treated versus untreated cells (dark colors depict Control and light colors LOAD astrocytes) or as LOAD effect, i.e., percent change in LOAD versus Controls (untreated cells are in dark and BHB-treated cells in light colors). **d** CyQuant fluorescence measurements on Control and LOAD astrocytes after conducting the Seahorse experiments in 3 mM and 12 mM BHB-treatment conditions. Results are plotted as percent change fluorescence comparing BHB-treated from untreated cells. Data are means +/- SEM from triplicate measurements in two repeat experiments. *$p < 0.1$; **$p < 0.05$; ***$p < 0.01$ using one-way ANOVA.

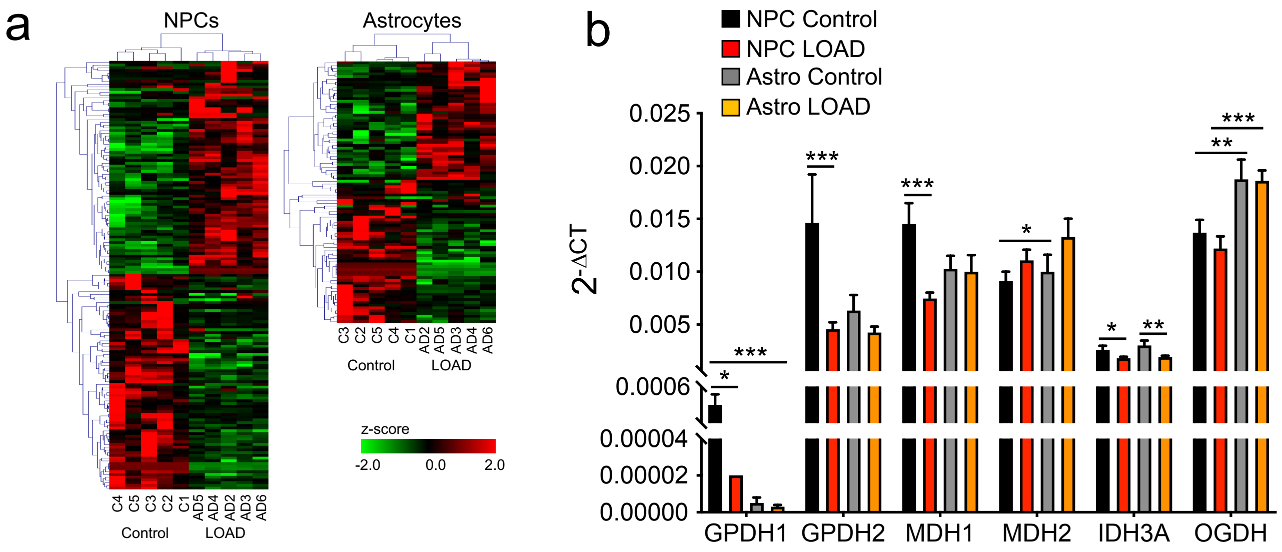

c

	NPCs				Astrocytes			
	qPCR		RNA-Seq		qPCR		RNA-Seq	
**Gene ID**	**Fold change**	**p value**	**Fold change**	**p value**	**Fold change**	**p value**	**Fold change**	**p value**
**NRK**	**-2.13**	**0.01**	**-4.13**	**0.00**	1.02	0.93	-1.20	0.45
**NMNAT2**	**2.79**	**0.06**	**3.08**	**0.00**	1.17	0.54	1.54	0.32
**NAMPT**	2.07	0.13	1.01	0.95	1.29	0.25	1.14	0.54
**GPD1**	**-12.89**	**0.02**	**-162.41**	**0.01**	NA	NA	1.12	1.00
**GPD2**	**-3.21**	**0.01**	**-3.01**	**0.00**	-1.49	0.16	-1.11	0.63
**MDH1**	**-1.95**	**0.00**	-1.07	0.76	-1.03	0.90	-1.10	0.63
**MDH2**	1.21	0.21	1.08	0.69	1.33	0.21	1.00	1.00
**IDH3A**	**-1.44**	**0.03**	-1.03	0.90	**-1.57**	**0.02**	-1.07	0.77
**OGDH**	-1.12	0.39	-1.12	0.57	-1.01	0.95	-1.04	0.84

Supplementary Fig. 11 LOAD and Control NPCs and astrocytes exhibit differences in their metabolic transcriptomes. a Unsupervised hierarchical clustering of genes from RNA-Seq experiments differentially expressed in LOAD (n = 5) or Control (n = 5) NPCs and astrocytes. Genes (rows) for individual samples (columns) were transformed to z-scores and plotted as heatmaps. **b** qRT-PCR results for GPDH1/2, MDH1/2, IDH3A, and OGDH, in LOAD (n = 9, red bars) and Control (n = 5, black bars) NPCs or LOAD (n = 9, orange bars) and Control (n = 5, grey bars) astrocytes plotted as $2^{-\Delta CT}$ values. Data are means +/- SEM from duplicate measurements in two repeat experiments. *p < 0.1; **p < 0.05; ***p < 0.01 using one-way ANOVA. **c** Comparison of fold changes from qRT-PCR with RNA-Seq data.

References

1. McPhie DL, Nehme R, Ravichandran C, Babb SM, Ghosh SD, Staskus A *et al.* Oligodendrocyte differentiation of induced pluripotent stem cells derived from subjects with schizophrenias implicate abnormalities in development. *Transl Psychiatry* 2018; **8**(1)**:** 230.

2. Sonntag KC, Ryu WI, Amirault KM, Healy RA, Siegel AJ, McPhie DL *et al.* Late-onset Alzheimer's disease is associated with inherent changes in bioenergetics profiles. *Sci Rep* 2017; **7**(1)**:** 14038.

3. Yoshimizu T, Pan JQ, Mungenast AE, Madison JM, Su S, Ketterman J *et al.* Functional implications of a psychiatric risk variant within CACNA1C in induced human neurons. *Mol Psychiatry* 2015; **20**(2)**:** 162-169.

4. Zhao J, Davis MD, Martens YA, Shinohara M, Graff-Radford NR, Younkin SG *et al.* APOE epsilon4/epsilon4 diminishes neurotrophic function of human iPSC-derived astrocytes. *Hum Mol Genet* 2017; **26**(14)**:** 2690-2700.

5. Li H, Li X, Ge X, Jia L, Zhang Z, Fang R *et al.* MiR-34b-3 and miR-449a inhibit malignant progression of nasopharyngeal carcinoma by targeting lactate dehydrogenase A. *Oncotarget* 2016; **7**(34)**:** 54838-54851.

6. Zhou C, Yu J, Wang M, Yang J, Xiong H, Huang H *et al.* Identification of glycerol-3-phosphate dehydrogenase 1 as a tumour suppressor in human breast cancer. *Oncotarget* 2017; **8**(60)**:** 101309-101324.

7. Zheng Y, Qu H, Xiong X, Wang Y, Liu X, Zhang L *et al.* Deficiency of Mitochondrial Glycerol 3-Phosphate Dehydrogenase Contributes to Hepatic Steatosis. *Hepatology* 2019; **70**(1)**:** 84-97.

8. Lee SM, Dho SH, Ju SK, Maeng JS, Kim JY, Kwon KS. Cytosolic malate dehydrogenase regulates senescence in human fibroblasts. *Biogerontology* 2012; **13**(5)**:** 525-536.

9. Snezhkina AV, Krasnov GS, Zaretsky AR, Zhavoronkov A, Nyushko KM, Moskalev AA *et al.* Differential expression of alternatively spliced transcripts related to energy metabolism in colorectal cancer. *BMC Genomics* 2016; **17**(Suppl 14)**:** 1011.

10. Di Stefano M, Galassi L, Magni G. Unique expression pattern of human nicotinamide mononucleotide adenylyltransferase isozymes in red blood cells. *Blood Cells Mol Dis* 2010; **45**(1)**:** 33-39.

11. Soncini D, Caffa I, Zoppoli G, Cea M, Cagnetta A, Passalacqua M *et al.* Nicotinamide phosphoribosyltransferase promotes epithelial-to-mesenchymal transition as a soluble factor independent of its enzymatic activity. *J Biol Chem* 2014; **289**(49)**:** 34189-34204.

12. Sociali G, Raffaghello L, Magnone M, Zamporlini F, Emionite L, Sturla L *et al.* Antitumor effect of combined NAMPT and CD73 inhibition in an ovarian cancer model. *Oncotarget* 2016; **7**(3)**:** 2968-2984.

13. Howe EA, Sinha R, Schlauch D, Quackenbush J. RNA-Seq analysis in MeV. *Bioinformatics* 2011; **27**(22)**:** 3209-3210.
